# Horizontal Gene Transfer Contributed to the Evolution of Extracellular Surface Structures: The Freshwater Polyp *Hydra* Is Covered by a Complex Fibrous Cuticle Containing Glycosaminoglycans and Proteins of the PPOD and SWT (Sweet Tooth) Families

**DOI:** 10.1371/journal.pone.0052278

**Published:** 2012-12-27

**Authors:** Angelika Böttger, Andrew C. Doxey, Michael W. Hess, Kristian Pfaller, Willi Salvenmoser, Rainer Deutzmann, Andreas Geissner, Barbara Pauly, Johannes Altstätter, Sandra Münder, Astrid Heim, Hans-Joachim Gabius, Brendan J. McConkey, Charles N. David

**Affiliations:** 1 Department Biologie II, Ludwig-Maximilians-University, Munich, Germany; 2 Department of Biology, University of Waterloo, Waterloo, Ontario, Canada; 3 Division of Histology and Embryology, Innsbruck Medical University, Innsbruck, Austria; 4 Institute of Zoology and Center for Molecular Biosciences Innsbruck, Innsbruck University, Innsbruck, Austria; 5 Department of Biology, University Regensburg, Regensburg, Germany; 6 Institute for Physiological Chemistry, Faculty of Veterinary Medicine, Ludwig-Maximilians-University, Munich, Germany; Iowa State University, United States of America

## Abstract

The single-cell layered ectoderm of the fresh water polyp *Hydra* fulfills the function of an epidermis by protecting the animals from the surrounding medium. Its outer surface is covered by a fibrous structure termed the cuticle layer, with similarity to the extracellular surface coats of mammalian epithelia. In this paper we have identified molecular components of the cuticle. We show that its outermost layer contains glycoproteins and glycosaminoglycans and we have identified chondroitin and chondroitin-6-sulfate chains. In a search for proteins that could be involved in organising this structure we found PPOD proteins and several members of a protein family containing only SWT (sweet tooth) domains. Structural analyses indicate that PPODs consist of two tandem β-trefoil domains with similarity to carbohydrate-binding sites found in lectins. Experimental evidence confirmed that PPODs can bind sulfated glycans and are secreted into the cuticle layer from granules localized under the apical surface of the ectodermal epithelial cells. PPODs are taxon-specific proteins which appear to have entered the *Hydra* genome by horizontal gene transfer from bacteria. Their acquisition at the time *Hydra* evolved from a marine ancestor may have been critical for the transition to the freshwater environment.

## Introduction

The freshwater polyp *Hydra* belongs to the phylum cnidaria and is thus a representative of one of the earliest pre-bilaterian metazoans. It has a simple body plan with an oral-aboral axis including a hypostome surrounded by tentacles, a gastric region and a peduncle with a basal disk. The tissue consists of two epithelia, the ectoderm and the endoderm, which are separated by the mesoglea, an extracellular matrix containing collagen and laminin [1].

The ectoderm of *Hydra* serves as an epidermis and forms the interface with the environment. In early electronmicroscopic studies of *Hydra* tissue, Lentz described the ectodermal extracellular surface as a “thin layer of homogeneous material of low density covered by a thicker feltwork of finely granular, fibrillar or filamentous material” with a thickness of 0.5 µm. In the basal disk it presents as a thick mucoid layer. Lentz also observed large mucous granules (0.5–1 µm) at the apical surface of ectodermal epithelial cells close to the plasma membrane, which probably secreted this material [2]. Based on its ultrastructural similarity with the extracellular surface coats of mammalian epithelia, which line the lumina of blood vessels, intestine, kidney glomerular vessels and other organs, this extracellular layer was later termed glycocalyx in cnidarian literature (see for example [3]–[5]).

Recent cryo-fixation EM images have revealed that the extracellular coat of *Hydra* has a complex layered structure ensheathing the animal like a cuticle [6]. Cuticles are well known structures among the invertebrates. Their size can vary from micrometers in some annelids up to several millimeters in decapods [7]. They are often hardened by incorporation of rigid proteins, such as specialised collagens and cuticlins in nematodes and annelids or chitin in arthropods. Cnidarian chitinous cuticles termed periderms are found in thecate hydroids. Calcareous cuticles are also known, e.g. from molluscs. These cuticles can thus withstand pressure from hydroskeletons or present themselves as rigid exoskeletons. The complex cuticle described here for the fresh water *Hydra* appears to be less rigid and is secreted directly by the ectodermal epithelial cells. Little is known about its molecular composition.

To investigate the molecular components of the cuticle, we first used the periodic acid-Schiff reaction (PAS) to verify the presence of carbohydrates. We then showed that chondroitin and chondroitin-6 sulfate could be washed off the cuticle with hypertonic salt solutions. By SDS-PAGE analysis of the salt wash we identified seven major protein bands. Three bands represented isoforms of PPOD (**p**utative **p**er**o**xi**d**ase) proteins [8], and four additional bands represented proteins containing only “sweet tooth” domains [9]–[11], which we have named SWT proteins. The SWT proteins were not investigated further but immunofluorescence imaging and immunogold EM with a PPOD-specific antibody confirmed that PPOD protein was localized in the cuticle and also in secretory granules of ectodermal cells underlying the cuticle.

PPOD 1 and 2 were originally identified as major components of secretory granules in ectodermal epithelial cells of *Hydra vulgaris* [8]. Because in peduncle cells these granules have peroxidase activity, it had been assumed that PPOD was the peroxidase responsible for this enzymatic activity. However, the expression patterns of PPOD genes did not correlate with peroxidase activities [12] and we show here that PPODs have two β-trefoil domains (arranged in tandem) typical of lectins found in both plants and animals [13], [14]. Experimentally, we demonstrate that PPOD can agglutinate erythrocytes and that this activity is inhibited by sulfated sugars, e.g the glycosaminoglycans heparin and chondroitin sulfate.

A search for PPOD homologs revealed bacterial homologs but no metazoan homologs suggesting that PPOD sequences entered the *Hydra* genome by horizontal gene transfer. Since PPODs are only present in *Hydra* species and not in closely related marine hydroids, we suggest that the acquisition of PPODs, which can modify the extracellular coat, may have been critical for the evolutionary transition from the marine to the freshwater environment.

In summary, this study aimed to get insight into the ultrastructure and molecular composition of the extracellular surface of *Hydra* polyps. It revealed a novel fibrous cuticle related to glycocalyx structures found on many cell types from amoebae to mammalian epithelial cells. Moreover, its outer layer contained glycosaminoglycans and *Hydra*-specific β-trefoil domain proteins capable of binding to chondroitin sulfate and thereby organising the structure of the cuticle.

## Materials and Methods

### Animals

*Hydra vulgaris* and *Hydra magnipapillata* were maintained in mass culture in *Hydra* medium as previously described [15]. Animals were fed *Artemia nauplii* several times per week.

### Isolation of Cuticula Components by Salt Wash

Animals were gently rocked for five minutes in *Hydra* medium containing 0.05, 0.1, 0.2 or 0.5 M sodium chloride (10 ml per culture dish containing approximately 2000 animals). After the animals had settled, the medium was taken off and centrifuged first at 3000×g, then at 13000×g. The proteins and carbohydrates in the supernatant were precipitated by adding two volumes of acetone and incubating at 4°C for at least seven hours. The sample was centrifuged at 4700×g, the supernatant discarded and the pellet resuspended in 1 M ammonium bicarbonate and lyophilized.

### SDS PAGE

Lyophilized samples were dissolved in reducing Laemmli buffer, heated to 92°C for at least five minutes and separated in 12.5% or 4–17.5% Laemmli gels.

### Immunoblotting

Samples for the immunoblotting were separated in a 12.5% SDS gel. The gel was cut into two parts, one was stained with Coomassie Blue, the other blotted onto an Immobilon PVDF membrane (Millipore). Both parts contained identical samples. The membrane was blocked over night at 4°C with 4% milk powder in PBS pH 7.5. Protein detection was carried out using a chicken-α-PPOD4 (Davids Biotechnologie, Regensburg) primary antibody (dilution 1∶1000 in PBST, 90 min at room temperature) and a donkey-α-chicken IgG coupled to IRDye 800CW (LI-COR Biosciences) secondary antibody (dilution 1∶5000 in PBST, 75 min at room temperature). The membrane was scanned on a LICOR Odyssey Imaging system (LI-COR Biosciences).

### PNGase F Treatment

For PNGase F treatment, lyophilized cuticule material obtained by salt washes was reduced with 1,4-dithioerythritol and carboxymethylated with sodium iodoacetate in a buffer containing 0.1 M Tris/HCl pH 8.2, 6.4 M GdmCl and 10 mM EDTA. After dialysis against 0.1 M ammonium bicarbonate, the samples were lyophilized and reconstituted in water (1/10 of the original volume). PNGase F (Sigma Aldrich) was added, and the sample and a control without the enzyme were incubated overnight at 37°C. The samples were lyophilized and analyzed in a 12.5% SDS gel.

### Chondroitinase ABC Digestion

Lyophilized cuticle material from salt washes was dissolved in chondroitinase buffer (50 mM Tris pH 7.4, 30 mM sodium acetate, 20 mM EDTA) and 7.5 mU of the enzyme was added. After incubation over night at 37°C, the samples were lyophilized.

### AMAC (2-aminoacridone) Labeling

The procedure for AMAC labeling by reductive amination was based on Deakin and Lyon [16]. 10 µl of 0.1 M AMAC in 85% DMSO/15% acetic acid were added to the sample in an aqueous solution of up to 30 µl. After 20 minutes incubation at room temperature, 10 µl of 1 M sodium cyanoborohydride were added and incubation was continued for at least 16 hours.

### PAGEFS

PAGEFS (polyacrylamide gel electrophoresis of fluorophore-labeled saccharides) of the labeled disaccharides from chodroitinase ABC digests was performed according to the protocol of Viola et al. [17]. This method separates AMAC labeled negatively charged saccharides by both, mass and charge. The buffer system is optimized for sulfated saccharides. Commercial disaccharides (chondroitin 0-, 4- or 6-sulfates; Sigma), that were HPLC purified after AMAC labeling, were used as references.

For MS analysis, bands from PAGEFS gels were cut out with a scalpel. The saccharides were extracted by shaking twice in water and once in 50% acetonitrile for at least 30 min each. The extracts were pooled, lyophilized and dissolved in a small volume of water. Saccharides were purified using C-18 ZipTips (Millipore) and eluted directly into the MALDI matrix.

### HPLC

AMAC-labeled disaccharides from chondroitinase ABC digests were separated by reversed-phase HPLC using a gradient of 20 mM ammonium acetate in water as buffer A and 20 mM ammonium acetate in 70% acetonitrile as buffer B (gradient: 10 to 40% B for 50 min, 40 to 100% B for 15 min, back to 10% B for 15 min). Fractions for MS analysis were collected by hand, lyophilized and directly dissolved in the MALDI matrix.

### Gel Filtration

Size determination of the CS chains was carried out by chromatography on a Sephacryl S-200 column (1.6×60 cm; GE Healthcare) with 0.2 M ammonium bicarbonate [18] as gel filtration buffer at a flow rate of 27 ml/h. Fractions were collected every 10 min. The column was calibrated using commercial dextrans (Sigma). Lyophilized salt wash was first digested with pronase in 50 mM ammonium bicarbonate/1 mM calcium chloride to remove proteins, lyophilized again, reconstituted in 0.2 M ammonium bicarbonate and applied to the column. The collected fractions were lyophilized and digested with chondroitinase ABC. After AMAC labeling, disaccharides were detected using PAGEFS.

### Protein Identification by Mass Spectrometry

For protein identification by mass spectrometry, gels were first rinsed with distilled water for several hours. Then protein bands were cut out of the gels using a scalpel and transferred into clean 2 ml tubes (Eppendorf). To remove substances interfering with trypsin digestion and/or mass spectrometry, the gel pieces were washed sequentially with 50 mM NH_4_HCO_3_, 50 mM NH_4_HCO_3_/acetonitrile (3/1), 25% acetonitrile, and 50% acetonitrile for 30 min each, respectively. After drying at room temperature, proteins were digested with 2 µg trypsin (sequencing grade, Roche) per 100 µl gel volume in 50 mM NH_4_HCO_3_ overnight at 37°C. Peptides were eluted by two extractions with 100 mM NH_4_HCO_3_, followed by one extraction with 50 mM NH_4_HCO_3_ in 50% acetonitrile. The combined extracts were lyophilized, resuspended in 50 µl H_2_O and lyophilized again to remove residual NH_4_HCO_3._

Mass spectrometry was done by MALDI-MS and -MS/MS on a 4800 Proteomics Analyzer running with the v3.5.3 4000 series explorer software (AB Sciex). For measurements the digests were dissolved in matrix solution (50% ACN/0.1% TFA containing 3.5 mg/mL α cyano-4-hydroxycinnamic acid, CHCA) and spotted onto target plates. MS was performed in positive ion reflector mode over the 800–4000 m/z mass range, typically with 1250 laser shots per spot and “plate default calibration” as specified by the manufacturer. MALDI-MS/MS was performed on the twelve most intense peaks, excluding trypsin autolysis peaks. The peptide ions were fragmented with 1 kV collision energy, and air as the collision gas, the masses of the fragment ions were calibrated using the “default calibration method”. Typically, MS/MS spectra were averaged over 4200 laser shots. The data were analyzed using GPS Explorer (v3.6. AB Sciex). Mascot (v2.1., Matrix Science) was used as a search engine to search a local copy (updated every three months) of the NCBInr protein data base or a *Hydra* specific database (Kyoto Bioinformatics Institute, www.genome.jp).

Mascot analyses were done using the following parameters: 50 ppm mass tolerance for peptide ions (MS mode) and 0.2 Da mass tolerance for fragment ions (MS/MS mode). Trypsin was selected as the cleavage enzyme, allowing for one missed cleavage. Methionine oxidation and cysteine modifications by iodoacetamide or acrylamide were allowed as variable protein modifications. The criterion for a reliable protein identification were Mascot scores >85 (NCBI) and >55 (*Hydra* database). These values define a cut off value where the probability of false positive identification is 5% [P<0.05; score: −10*log(P); P is the probability that the observed match is a random event]. Typically the proteins were identified unambiguously with Mascot scores above 150.

### MS of Carbohydrates

As for analysis of proteins, mass spectrometry of carbohydrates was done by MALDI-MS and -MS/MS on a 4800 Proteomics Analyzer running with the v3.5.3 4000 series explorer software (AB Sciex).

For AMAC-labeled disaccharides the following matrices were used:

HPLC-purified samples: Mixture 1∶1 of 3.4 mg/ml CHCA in 50% acetonitrile containing 10 mM sodium acetate and 6.67 mg/ml SDHB (9∶1 mixture of DHB (2,5-dihydroxybenzoic acid) and 5-methoxysalicylic acid; Sigma) in 50% acetonitrile with 10 mM sodium acetate
Gel eluted samples purified via ZipTips: Mixture 1∶1 of 3,4 mg/ml CHCA in 50% acetonitrile with 0.1% TFA and 6.67 mg/ml SDHB in 50% acetonitrile containing 10 mM sodium acetate

MS/MS spectra were evaluated manually.

### Immunofluorescence Staining with Anti-PPOD4 Antibody

Anti-PPOD4 antibody was raised in a chicken at Davids Biotechnology, Regensburg, Germany by immunisation with purified His-tagged recombinant PPOD 4 protein (see below).

*Staining of apical granules:* Animals were relaxed in 2% urethane in *Hydra* medium and fixed 1 h at room temperature with Lawdowsky’s fixative (48% ethanol, 3.6% formaldehyde and 3.8% acetic acid in water). After three washes with PBS, the animals were permeabilized with 0.5% Triton X-100 in PBS for 15 min, blocked with 0.1% Triton-X-100, 1% BSA (w/v) in PBS for at least 20 min and incubated with anti-PPOD4 and anti-GFP (Roche) antibodies overnight at 4°C. The next day animals were washed three times with PBS and incubated for 2 h with anti-rabbit-Cy3 (GE Healthcare) and with anti-mouse-Alexa488-conjugated (Invitrogen) second antibodies. The animals were washed again three times with PBS, counterstained for DNA with DAPI (Sigma, 1 µg/ml) and mounted on slides with Vectashield mounting medium (Vector Laboratories).

*Staining of extracellular components:* Transgenic animals that express eGFP in ectodermal epithelial cells were used [19]. Polyps were treated as above except that fixation was with 2%PFA in *Hydra* medium and the fixed tissue was not permeabilized.

*Salt treatment of whole animals:* Prior to antibody staining animals were treated with 500 mM NaCl in *Hydra* medium for 5 min at room temperature.

### Electron Microscopy

Cryofixation and conventional chemical fixation of *H. vulgaris* and *H. magnipapillata* for transmission electron microscopy (TEM) and scanning electron microscopy (SEM) were performed essentially as described [6]. Briefly, the following TEM-protocols were used: (i) high-pressure freezing (HPF), followed by freeze-substitution (FS) with acetone (containing 0.5% OsO4 and 0.1% uranyl acetate) and Epon embedding (for morphology and cytochemistry), (ii) HPF, FS with pure acetone and LR-white embedding (for immuno-EM and cytochemistry), (iii) chemical fixation with a mixture of 3% aqueous glutaraldehyde and 1% OsO_4_ in ∼0.05 M phosphate buffer (modified after [20]), supplemented with 0.15% ruthenium red, followed by 2% aqueous OsO4 (also containing 0.15% ruthenium red) and Spurr’s-embedding (ruthenium red (ammoniated ruthenium oxychloride) generally improves the preservation of extracellular matrix/cell coats [21]. For high-resolution SEM, *Hydra* samples were high-pressure frozen, fractured, freeze-substituted with acetone (containing 0.5% OsO4), followed by critical-point drying, and sputter coating.

Indirect immuno-gold labelling was performed on LR-White sections [22] by using the chicken anti-PPOD4 antibody described here and rabbit-anti-chicken colloidal gold (10 nm; British Biocell, Cardiff, U.K.). Omission of primary antibody as a control yielded negligible background labelling.

Cytochemical detection of PAS-positive carbohydrates was carried out according to [23]. Sections from cryofixed samples embedded in Epon or LR-White were exposed to periodic acid (PA: 1%, 2–24 hrs.), thiocarbohydrazide (TCH: 0.2%, 2–24 hrs.) and silver-proteinate (SP: 1%, 30 min.). Controls included omission of PA or blocking of aldehydes generated by PA-treatment (with 1% sodium borohydride, 4 min.).

Semithin sections were stained with toluidine blue, ultrathin sections were (optionally) contrasted with uranyl and lead salts. Samples were viewed with a Eclipse 80i (from Nikon, Tokyo, Japan), a CM120 (from Philips/FEI, Eindhoven, The Netherlands), with a LIBRA 120 or a Gemini-DSM 982 (both from ZEISS, Oberkochen, Germany).

### In Gel Peroxidase Assay

Vesicles were isolated from *Hydra* tissue by homogenisation and differential centrifugation. They were dissolved in 250 mM sucrose, 5 mM Tris/HCl pH 7.5, 4 mM pefabloc, 1 mM vanadate, 2 mM MgATP, 10 µg/ml antipain and separated in 12% LDS-gelelectrophoresis. The gel was incubated over night with 0.06% diaminobenzidine, 0.03% H_2_O_2_ in 100 mM citrate buffer pH 5.0 was added and the reaction was stopped by several washes in H_2_O [24].

### Expression of PPOD4 in E. coli

The PPOD4 coding sequence (amino acids 21–290) was amplified from cDNA and cloned into the vector pRSETA (Invitrogen). Lysis of bacteria expressing an N-terminally His-tagged protein was carried out using a French press. Soluble protein was bound to a Ni-NTA agarose-column and eluted with imidazole. The bound protein was dialysed against 25 mM HEPES, 1 mM DTT 20% glycerol and used for antibody preparation in a chicken as described above (Davids Biotechnology, Regensburg, Germany) and for haemagglutination experiments.

### Haemagglutination

Assays were perfomed with trypsinized, glutaraldehyde-fixed rabbit erythrocytes in microtiter plates. PPOD protein was added at the indicated concentrations. Diverse sugars and proteoglycans were used as test compounds as described in detail previously [25], [26].

### Bioinformatic Analysis of PPODs

The structure of PPOD4 was modeled using the Phyre algorithm [27]. The Phyre result using the full length PPOD4 amino acid sequence suggested a domain architecture based on two consecutive β-trefoil folds. Based on this information, domain 1 (repeats 1–3) and domain 2 (repeats 4–6) were then modeled separately, which accounted for alignment errors due to the highly repetitive sequence. Internal repeats were also predicted at the sequence level using the RADAR algorithm [28] and mapped onto the structural model. For consistency, when compared to other β-trefoil structures, secondary structures were assigned to all structures using STRIDE [29].

A BLAST search was performed using PPOD4 domain 1 (residues 1–161) as a query sequence. All sequences scoring with E-value ≤0.05 that aligned to ≥80% of the query sequence, were collected and realigned using MUSCLE [30]. Following removal of poorly aligned regions, 156 remaining polymorphic sites were used for phylogenetic tree construction. A maximum-likelihood tree was generated using PhyML [31] using the WAG model with 4 rate categories, and including estimation of the proportion of invariable sites and gamma distribution parameter. Clade support was evaluated using aLRT statistics.

The domain architectures of the full-length proteins were retrieved from the NCBI conserved domain database (CDD). The NCBI-annotated ‘fascin’ domains were removed and replaced with locations of PPOD internal repeats predicted separately using HMMer with an HMM-trained on individual PPOD repeats. This was used to visualize the internal repeat architecture but was not used in analysis.

The conservation logo of individual repeats was constructed using webLogo and 24 sequences of individual repeats associated with the 8 β-trefoil domains from PPODs 1–4. The solvent-accessibility was measured using the method of [32] using the binding-site associated repeat from PPOD4 (domain 2).

## Results

### Structure and PAS-cytochemistry of the *Hydra* Cuticle

**Figure 1:**
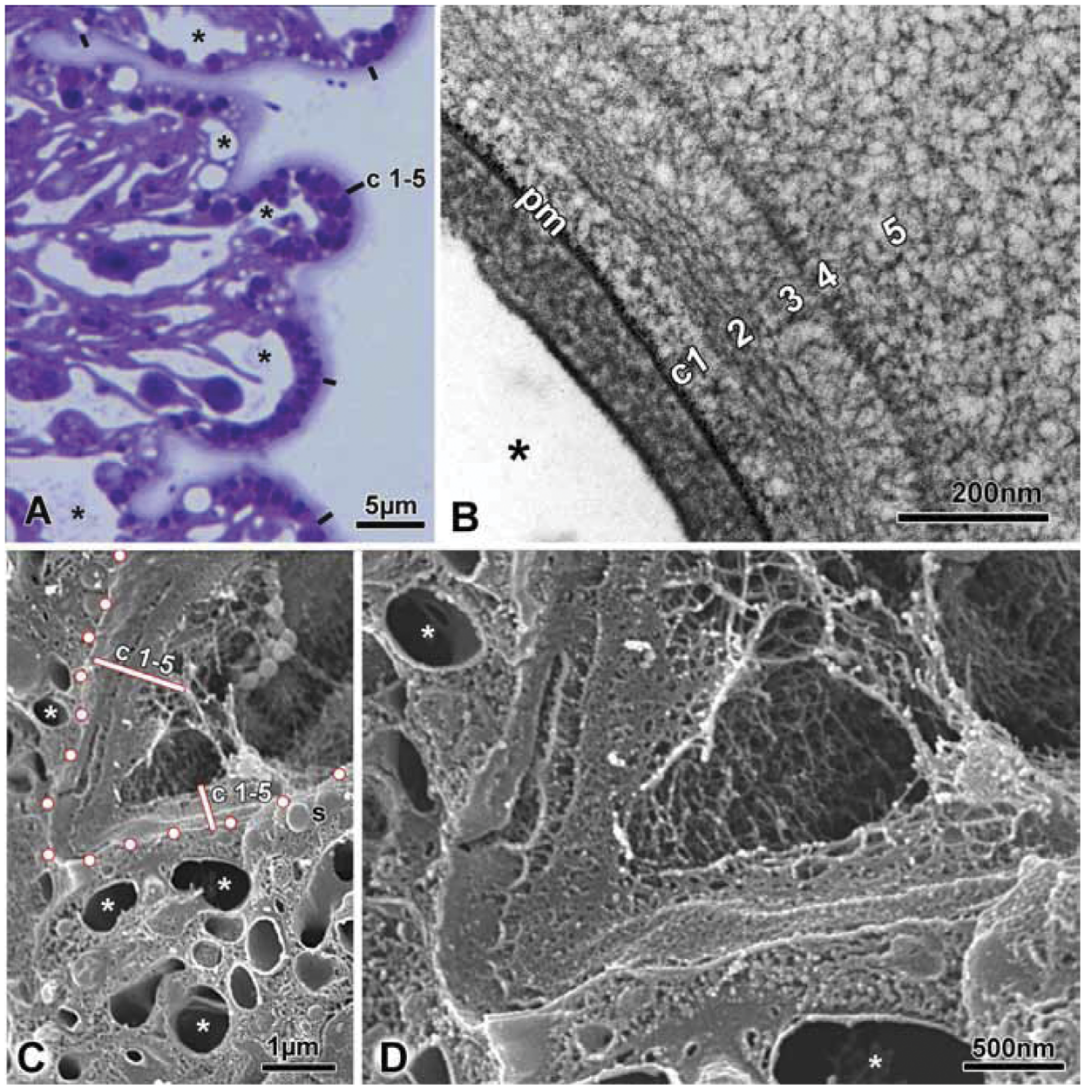
Light and electron microsopic images of cryo-fixed *Hydra* cuticle. A: Overview of 0.5 µm semi-thin Epon section stained with the basic dye toluidine blue. The cuticle (c1–5) covering the whole *H. vulgaris* polyp is clearly visible. Asterisks mark the pleiomorphic intra−/intercellular vacuolar system. Scale bar: 5 µm. B: Transmission EM reveals the fibrous appearance of layer c2 and the periodically arranged rod-like substructures forming layer c4. Plasma membrane marked by (pm), asterisk marks a vacuole. Note the strong contrast of all 5 cuticle layers and the plasma membrane obtained with the PAS-reagent (see also Fig. 2). Scale bar: 200 nm. C: Scanning EM of freeze-fractured *H. magnipapillata* ectodermal epithelium reveals the overlying cuticle (c1–5) as a bulky structure (note: individual cuticle layers cannot be distinguished in the SEM-samples). Dots mark the apical plasma membrane of the epithelium, asteriks mark the vacuolar system, secretory granules are indicated by “s”. Scale bar: 1 µm. D: Magnified view of Fig. 1C. Scale bar: 500 nm.

Recent work using cryo-based fixation methods (high-pressure freezing and freeze-substitution; termed cryo-fixation in the following) has shown that this method stabilises the ultrastructure of the *Hydra* cuticle very effectively [6]. Samples prepared in this way show the cuticle as a homogeneous coat covering the whole ectodermal surface (Fig. 1A). Transmission EM on thin sections shows five distinct layers in the cuticle (c1– c5) made up, at least partially, of fibrous material (Fig. 1B). Some of the fibres appeared to be regularly oriented, i.e. parallel or perpendicular to the cell membrane (c2, c4). The c4 layer is narrow and consists of heavily stained short rods, which appeared in many cases to form the base of long fibrous elements extending as an irregular network into the surrounding medium. This loose meshwork constitutes the c5 layer. The whole cuticle extends up to 1.5 µm from the cell surface. Scanning EM of freeze-fractured ectodermal epithelium confirmed the robust, fibrous nature of the cuticle (Fig. 1C, D). Although the individual layers could not be clearly distinguished in freeze-fractured samples, the outer surface corresponding to layer c5, clearly consisted of a porous meshwork of fibres. Frequently, bacterial cells and *Naegleria* amoeba were seen attached to the surface of this meshwork (data not shown), suggesting that it represents a barrier separating microbes from the plasma membrane.

**Figure 2:**
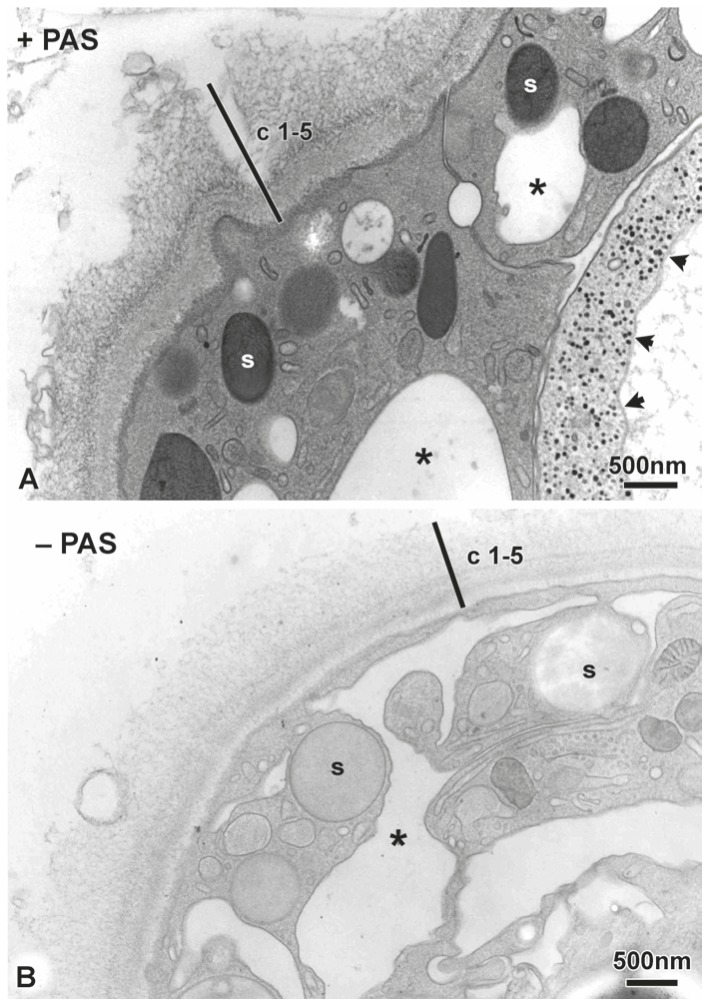
PAS cytochemistry of *Hydra* cuticle. Periodic acid-thiocarbohydrazide-silver proteinate staining was performed on Epon sections from *H. vulgaris*. A: Cuticle layers c1–5 react positively as do apical secretory granules (s) and glycogen particles (arrow-heads) in a neighbouring nematocyst; the asterisk marks a vacuole. Scale bar: 500 nm. B: Negative control for the PAS-reaction (omission of periodic acid oxidation); faint unspecific staining results from binding of thiocarbohydrazide to the osmium tetroxide used for freeze-substitution. Scale bar: 500 nm.

To determine whether the cuticle contains sugars, we carried out PAS-cytochemistry at the EM level. The result in Fig. 2A, B shows that all layers of the cuticle are strongly PAS reactive (see also Fig. 1B), as are the plasma membrane, the large secretory vesicles under the apical surface and glycogen particles.

### Hypertonic Salt Washes Release Carbohydrates and Proteins from the Cuticle

Sugar coats on cell surfaces constitute a very special microenvironment. In particular, negatively charged sugars and proteoglycans have the capacity to act as ion-exchangers and exposing such structures to high salt concentrations is expected to release non-covalently bound components such as heparan sulfate or chondroitin sulfates and lectins specific for these glycosaminoglycans [26], [33], [34]. To investigate how stable the *Hydra* cuticle is and whether it contains non-covalently linked components, we treated polyps briefly with 0.1 and 0.2 M NaCl solutions. The treatment did not injure the polyps and essentially all of them recovered after a few minutes. Cytoplasmic proteins (e.g. 14-3-3 proteins) were not released by the salt treatment (data not shown). EM analysis of the cuticle layer showed that the salt wash caused swelling and extensive extraction of the cuticle (especially layer c5), but not complete dissolution of the other cuticle layers (Fig. S1). To identify components released by the salt wash, we looked for glycosaminoglycans and proteins.

#### Chondroitin and chrondroitin sulfate in the salt wash

**Figure 3:**
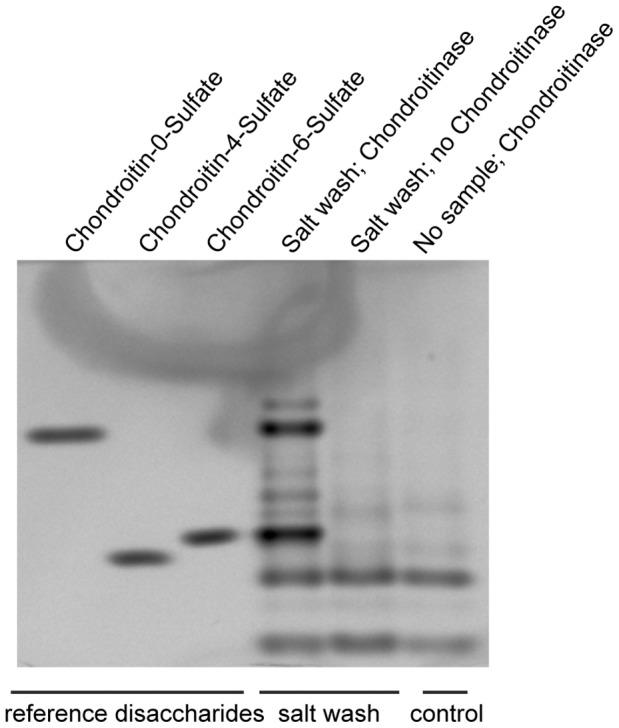
Chondroitinsulfates in hypertonic salt wash. The salt wash was digested with chondroitinase and the resulting disaccharides labeled with AMAC and separated by PAGEFS. The left three lanes are standard disaccharides.

In a previous study chondroitin sulfate and heparan sulfate were identified in homogenates of *Hydra* tissue, with chondroitin sulfate being the major glycosaminoglycan [18]. Thus, we looked for the presence of glycosaminoglycans in the salt wash by treatment with chondroitinase. Following enzymatic digestion, the resulting disaccharides were labeled with the fluorophore AMAC and separated by gel electrophoresis (30% polyacrylamide gel without SDS). Disaccharides of unsulfated chondroitin and chondroitin 4- and 6-sulfate could be separated under these conditions. Fig. 3 shows that the cuticle contains predominantly unsulfated chondroitin and chondroitin-6-sulfate disaccharides. To confirm the structures, disaccharides were either eluted from the gels or purified from the reaction mixture by reverse-phase chromatography and subsequently analyzed by mass spectrometry determining masses in the MS mode and characteristic fragment spectra in the MS/MS mode.

Interestingly, no chondroitin 4-sulfate disaccharides were observed in chondroitinase digests of the salt wash, although these are present in glycosaminoglycans isolated from whole animals ([18], Deutzmann unpublished). We also tried to identify the nature of the core proteins, since chondroitin sulfate chains, are commonly synthesized in protein-bound form. After chondroitinase digestion no new protein bands were observed in SDS gels of salt washes. Furthermore, digestion with the unspecific protease Pronase did not alter the size of the glycosaminoglycan chains (not shown). These experiments indicate the absence of large core proteins, but do not rule out that small peptides could still be attached to the sugar residues. Using a Sephacryl-S200 column (calibrated with dextran standards) the mass of the chondroitin-sulfate chains was determined to be in the range between 20 and 80 kDa, with a maximum at about 40 kDa (not shown).

#### Proteins in the salt wash

**Figure 4:**
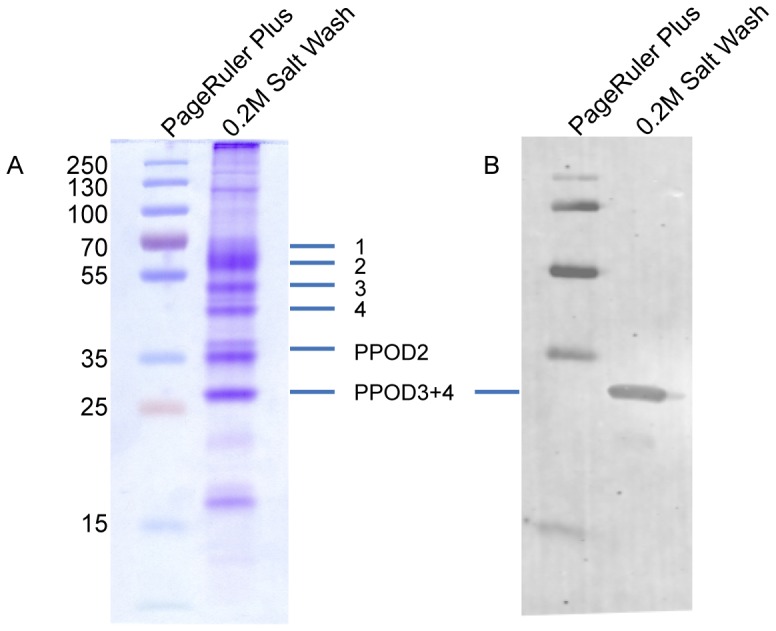
Soluble protein content of hypertonic salt wash. A: SDS-PAGE gel of 0.2 M NaCl wash stained with coomassie. Major bands were excised and identified by mass spectrometry. Positions of the PPOD 2, 3 and 4 bands are indicated. Bands 1–4 represent members of SWT protein family. See text for details. B: Immunoblot for this gel stained with anti-PPOD4 antibody.

To identify proteins present in the cuticle, we separated the salt wash on an SDS-PAGE gel. The Coomassie-stained gel revealed seven major protein bands ranging in mass from 27 to 70 kDa (Fig. 4A). These bands were excised from the gel, the proteins digested with trypsin and analysed by mass spectrometry. The results indicated that three bands contained members of the PPOD family. The 27 kDa band contained PPODs 3 and 4, while the bands at 35 and 37 kDa both contained PPOD2. Mascot Scores for the PPODs were between 311 and 599 and allowed unambiguous identification of these proteins, since the threshold for 95% significance was a score of 85 (Table 1).

**Table 1:** Identification of PPOD proteins in the SDS-PAGE gel by mass spectrometry.

PPOD	Hma2	XP	Mascot score (combined Ms+Ms/Ms)	Sequence coverage*	PPOD isoform specific peptides, identifiedby MS/MS**	Peptide Ms/Ms scores
PPOD1	201736	XP_002170748.1	311	36% (8/3)	none	
PPOD2	211682	XP_002161691.1	599	53% (14/6)	90–105: FVTAVNTDTNLLIANK	94
					133–148: LVTAEEAGNKPLTANR	122
					195–216: AQIGPWETFEISFSDSQTLTLK	115
					275–281: SYLIANR	43
					282–289: DNADIWER	65
PPOD3	205478	XP_002155912.1	435	41% (9/5)	41–56: FVCAENVGSQPLIANR	105 (evaluated manually)
					261–276: LVTAENAGHDNLIANR	
PPOD4***	211683	XP_002161930.1	451	55% (13/5)	Common to both PPOD4-species:	
					56–67: DSIGLWETFEIR	118
					68–77: FTDAQTFVLK	78
					276–283: DKADIWER	60
					Unique:	
					100–120: DQATVFETFTLVPSFGTFGFK	61
		XP_002159894.1	427	45% (11/4)	Common to both PPOD4-species:	
					56–67: DSIGLWETFEIR	118
					68–77: FTDAQTFVLK	78
					276–283: DKADIWER	60
					Unique:	
					217–232: LVCAENSGQFPLIANR	90
PPOD5	no Hma	XP_002157652			none	

* Numbers in parentheses refer to numbers of peptides identified by MS-spectra and MS/MS spectra respectively.

** Numbers give the position of the peptides in the protein sequence.

*** There are two slightly different PPOD4 gene models (XP_002161930 and XP_002159894) based on NCBI annotation of the *Hydra* genome. Unique peptides corresponding to both gene models were found in the 27 kDa band. By comparison, only one PPOD4 gene model (Hma2.211683) corresponding to XP_002161930 is found in the *Hydra* genome browser (see [11] for details of two genome assemblies).

The bands numbered 1–4 in Fig. 4 contained members of a large family of proteins, which have two tandem copies of the sweet tooth (SWT) domain. We call these proteins SWT proteins, since they consist only of sweet tooth domains. The SWT domain was first identified in the extracellular domain of a receptor tyrosine kinase in *Hydra* [9] and later in a signal peptide screen for secreted proteins [10]. The *Hydra* genome encodes 22 SWT proteins with two sweet tooth domains (Table 2) [11]. Although the SWT proteins have 370–390 amino acids and a predicted molecular mass of 38 kDa, they gave rise to bands at 45, 50, 60 and 70 kDa in the SDS gels suggesting that they are post-translationally modified. Mass spectrometry of the proteins in bands 1–4 revealed multiple peptides with highly significant MS/MS scores. Most peptides, however, occur in several different members of the SWT family, because the SWT sequences are highly similar to each other. Hence it was not possible to assign single SWT proteins to each band. Rather it appears that each band contains two to three closely related SWT proteins (Table 3).

**Table 2:** Annotated SWT-Proteins containing two sweet tooth domains.

SWT Protein	NCBI annotation
1	XP_002166394
2	XP_002166371
3	XP_002161900
4	XP_002161876
5	XP_002164311
6	XP_002165870
7	XP_002163192
8	XP_002155918
9	XP_002168626
10	XP_002164861
11	XP_002154912
12	XP_002162962
13	XP_002156093
14	XP_002164279
15	XP_002154224
16	XP_002169694
17	XP_002160782
18	XP_002163701
19	XP_002169658
20	XP_002154560
21	XP_002167917
22	XP_002161707

There are additional SWT family members with only a single SWT domain and family members with more than two SWT domains.

**Table 3:** SWT (sweet tooth) proteins identified in the salt wash by MS/MS.

peptide identifiedby MS/MS	MS/MS-score	sweet tooth protein no.									band no. inSDS-gel
		3	4	5	7	8	9	12	13	18	
TEFQIVTSINENPNR	70		X	X							1
LHVVFIPHHNFNSEPLPLNK	131		X	X							1
QVYIDELPLNEWTK	81	X	X	X							1
SVQQNELVAAIPVLAK	67	X	X								1
LNSFSDGYR	57	X	X	X				X			1
SFDISFDL	54	X	X	X				X			1
VVISQQR	32		X	X							1
TEFQIVTSINENPNR	79		X	X							2
LNSFSDGYR	65	X	X	X				X			2
VVISQQR	38		X	X							2
LNSFSDGFR	53									X	2
VVDNYVFTIK	52									X	2
TPALWFRPYESNYK	52									X	2
VFASDPWYPSQDGSIK	75				X	X	X		X	X	2
LNSFSDGFR	60									X	3
QHAESGR	58				X	X	X				3
VSFDLKPK	30				X		X				3
IPGIWIFDR	35				X		X				3
LEQSFDIYFDLK	97				X	X	X		X		3
VYAGDPWYEAQDGSIK	103				X	X	X		X		3
NEFFNSKPLQR	75					X			X		4
LEQSFDIYFDLK	91				X	X	X		X		4
YFVNNEVEETLKK	104					X					4
TPALWLRPYEPNYR	60					X					4
VYAGDPWYEAQDGSIK	96				X	X	X		X		4
QINIDALPLNVWTNVVISQQR	165					X					4
GFNSVIHLTIGEDNSK	148								X		4
NFVYNEVEETLKK	50									X	4

In conclusion, the *Hydra* cuticle contains non-covalently bound material including chondroitin and chrondroitin 6-sulfate and proteins from the PPOD and SWT families. We have focussed on PPOD proteins and show that they fulfill the criterion for lectin but not peroxidase activity as previously assumed. The SWT proteins have not been further analyzed here.

### PPOD Isoforms

The *Hydra* genome encodes five isoforms of PPOD (see Table 1). PPOD2, 3 and 4 were identified by mass spectrometry in the salt wash (Fig. 4). In SDS-PAGE gels PPOD3 and PPOD4 migrated at an apparent molecular weight of 27 kDa in agreement with the predicted size of the mature proteins. PPOD2 ran, by contrast, at a higher apparent molecular weight. This could be due to glycosylation, since PPOD2, in contrast to PPOD3 and 4, has a conserved N-glycosylation site at Asn-52. Treatment of the salt wash with PNGase F (an enzyme that splits the bond between Asn and the first GlcNAc of the oligosaccharide chain thereby converting Asn to Asp) caused the PPOD2 bands to co-migrate with PPODs 3 and 4 at 27 kD in SDS gels (data not shown). Furthermore, mass spectrometry of this band yielded the PPOD2-specific peptide FVCAEK***D***GSEPLIANK with an aspartate at the position of asparagine confirming that PPOD2 was deglycosylated by treatment with PNGase F.

We conclude that PPOD2, 3 and 4 are the major isoforms of PPOD present in salt washes. PPOD2 is N-glycosylated on Asn-52. Our failure to detect PPOD1 could have been due to the fact that this isoform is expressed only in foot cells and may constitute a minor fraction of the extracellular PPOD proteins [8]. The gene model for PPOD5 is incomplete and there are no ESTs. Thus it is unclear whether the gene encoding this isoform is expressed.

### Localisation of PPOD in *Hydra* by immunohistochemistry and Immuno-EM

We immunised a chicken with full-length recombinant PPOD4 produced in *E. coli*. The resulting antibody recognised both the recombinant PPOD4 protein in bacterial lysates (Fig. S2) and endogenous PPOD4 in lysates of *Hydra* tissue after SDS-PAGE and immunoblotting (Fig. 4B). The latter gave a single band at the expected molecular weight of PPOD4 (27 kDa). The PPOD2 bands at 35 and 37 kDa were not recognized by the antibody (Fig. 4B).

**Figure 5:**
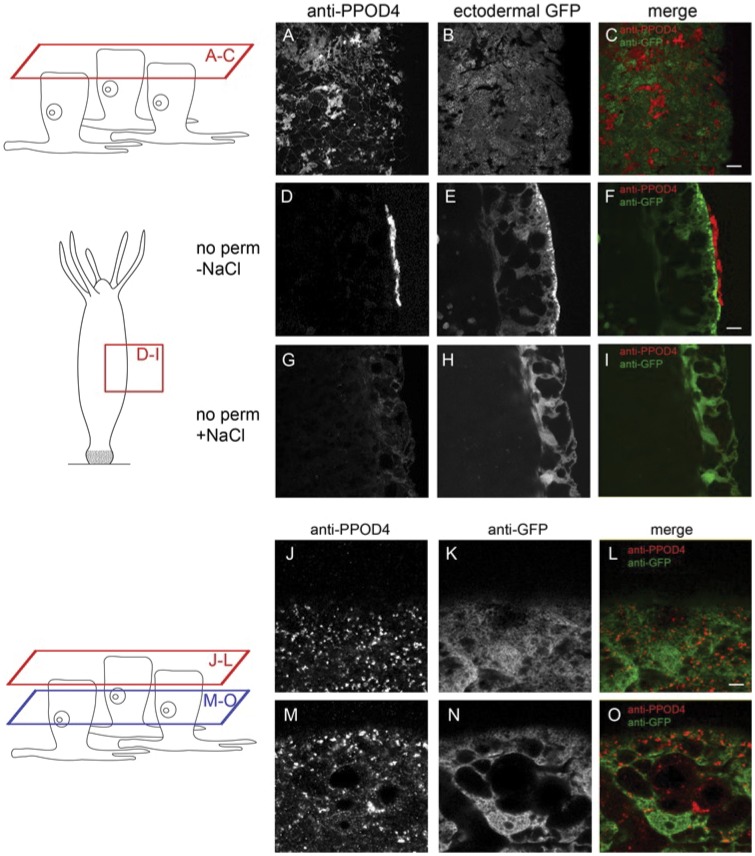
Immunofluorescence staining of *Hydra* polyps with anti-PPOD4 antibody. Polyps were fixed with PFA (A-I) or Lawdowsky’s fixative (J–O), stained with anti-PPOD4 or anti-GFP antibody and examined in the confocal microscope. Schematic diagrams indicate the positions of the optical sections shown. Polyps in A-I were not permeabilized and show staining of the extracellular surface. Polyps in J-O were permeabilized to permit staining of intracellular vesicles. See text for details.

To localize PPOD4 in tissue we stained *Hydra* whole mounts with the anti-PPOD4 antibody after fixation with PFA and without permeabilisation. In order to precisely position PPOD staining relative to the cell boundary we used animals expressing GFP in ectodermal epithelial cells [19]. Fig. 5A–C shows that the PPOD-positive material was clearly extracellular and appeared patchy, indicating that it was probably fragile and sensitive to the staining procedure. When such stained patches were imaged at higher magnification and in profile, it became clear that they are fragments of a poorly fixed but presumably uniform sheet of extracellular material covering the ectoderm (Fig. 5D–F). In order to confirm that salt treatment removes PPOD from the cuticle, we also stained animals after the salt wash. This, in complete agreement with our previous results, removed the extracellular PPOD-signal (Fig. 5G–I).

We next fixed animals with Lawdowsky’s fixative, which preserves secretory vesicles better than PFA fixation, and included permeabilisation of the tissue to allow access of the antibody to intracellular structures. We detected numerous antibody-stained secretory vesicles located close to the apical surface of ectodermal epithelial cells (Fig. 5, J–L and M–O). The stained vesicles were approximately 1 µm in diameter.

**Figure 6:**
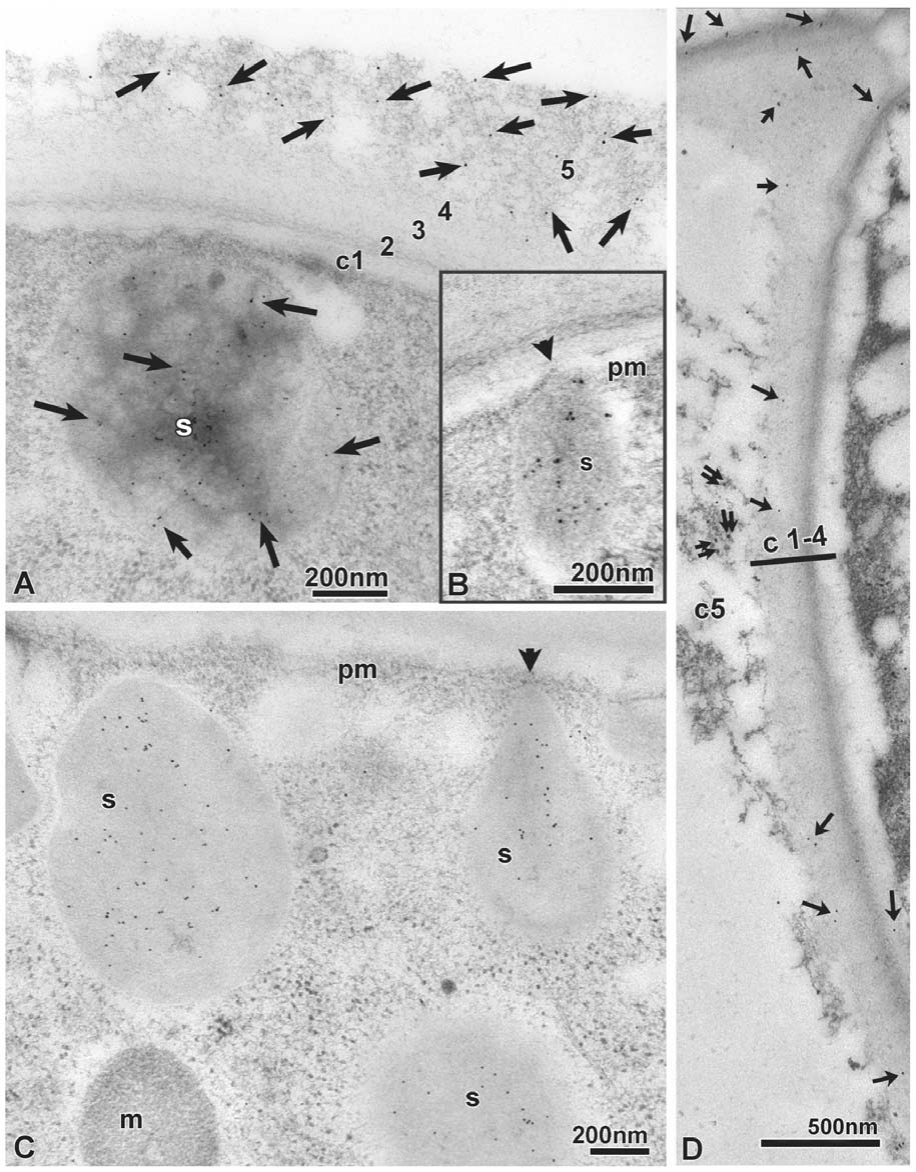
Immuno-EM localisation of PPOD. A: Immunogold labeling of PPOD (visualised by anti-chicken 10 nm colloidal gold) in the cuticle layer c5 and in subapical secretory granules (s) of ectodermal epithelial cells. Freeze-substitution with pure acetone followed by LR-white embedding. Scale bar: 200 nm. B: PPOD-positive secretory granule (s) in contact (arrow-head) with the plasma membrane (pm). Scale bar: 200 nm. C: Subapical secretory granules (s) are all PPOD-positive. A mitochondrium is lettered with (m), the plasma membrane with (pm). Scale bar: 200 nm. D: Scarce PPOD-immunogold-labelling can also be seen throughout cuticle layers c2–4 (marked by arrows), in addition to PPOD-labelling of layer c5 (double arrows). Scale bar: 500 nm.

Finally, to determine which layers of the extracellular cuticle contain PPOD, we carried out immunogold labelling on thin sections of cryo-fixed tissue. PPOD was abundant in layer c5 of the cuticle (Fig. 6A). Secretory vesicles under the apical surface of ectodermal cells were also clearly positive, and some showed close contact to the plasma membrane (Fig. 6B, C). Occasional gold particles were also seen in the other layers (Fig. 6D).

In summary, these results suggest that PPOD is secreted into the cuticle from secretory vesicles beneath the apical surface of ectodermal epithelial cells and is mainly localised in the outermost layer of the cuticle together with soluble chondroitin sulfate. These data prompted us to re-investigate the biochemical function of the PPOD proteins.

### PPOD does not have Peroxidase Activity

In order to clarify whether PPOD has peroxidase activity as previously suggested [8], we performed an in-gel peroxidase assay on recombinant PPOD protein by exposing LDS-gels to diaminobenzidine and hydrogen peroxide (Fig. S3). Recombinant PPOD had no detectable peroxidase activity. We then tested endogenous PPOD isolated from secretory vesicles. Endogenous PPOD was identified on the immunoblot of this gel at the same position as recombinant PPOD and had no peroxidase activity, although the vesicle fraction did contain a peroxidase activity at a different position in the gel, an internal positive control. We conclude from these experiments that both endogenous and recombinant PPOD lack peroxidase activity. In order to establish a biochemical function for PPOD, we next turned to structural modeling.

### Structural Modeling of *Hydra* PPOD

#### PPOD has two β-trefoil domains

The structure of PPOD4, the representative PPOD used in this study, was computationally calculated based on sequence homology to known structures in the Protein Data Bank (PDB) using Phyre [27]. Phyre detected two domains in PPOD4, each adopting a β-trefoil fold. The top-scoring PDB matches used by Phyre to model the PPOD structure were the β-trefoil domains of the eukaryotic actin-bundling protein fascin (PDB ID 1DFC,) with E-value <1e-09 and estimated precision of 100% [35]. This relationship was verified by a PSI-BLAST search, in which 1DFC was the top PDB match to the PPOD4 query detected in the second iteration and had 21% sequence similarity and an E-value <0.006. The distant but still significant similarity between PPODs and fascins is also consistent with the NCBI’s automated annotations using Conserved Domain Database models, which currently annotate PPODs as containing two consecutive fascin domains. The lower-scoring hits detected by Phyre were also all β-trefoil structures. The same result was obtained using other PPOD family members.

**Figure 7:**
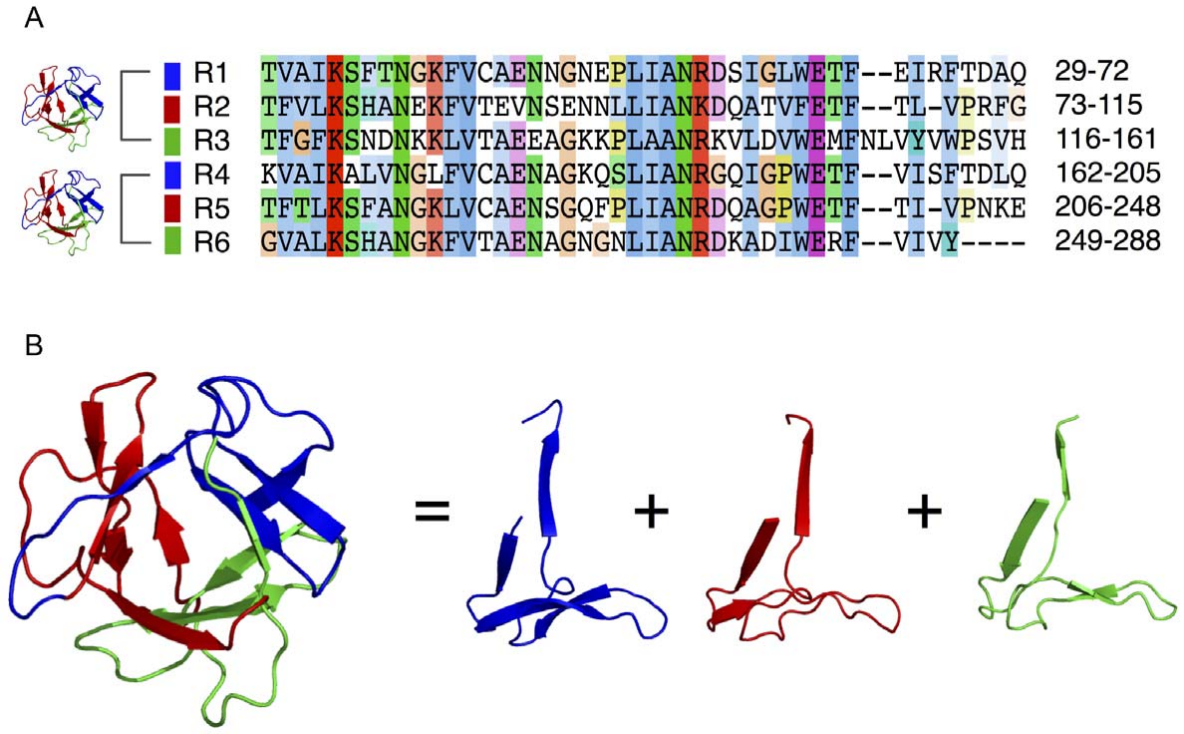
Internal repeats and three-dimensional structure of *Hydra* PPOD. A) Alignment of six internal repeats detected within the PPOD4 sequence using the RADAR algorithm. B) Structural model of a single β-trefoil domain in PPOD4 as inferred by Phyre. Three internal sequence repeats (coloured ribbon models) correspond to three repeated supersecondary structures that form a single β-trefoil fold.

The β-trefoil fold is composed of three internal repeats, each encoding a beta-beta-beta-loop-beta supersecondary structural element [36]. Consistent with previous results [8], six repeats were detected within PPODs using the RADAR [28] repeat detection tool (Fig. 7A). The predicted repeats encompass the full-length PPOD4 sequence with the exception of the first ∼20 residues, which contains a signal peptide according to TargetP [37]. When mapped onto the predicted structure of PPOD4, each sequence repeat was found to correspond to a single structural repeat element (beta-beta-beta-loop-beta) (Fig. 7B). Thus, six of the repeats form two consecutive β-trefoil domains in PPODs.

#### PPODs are related to bacterial domains associated with glycoside hydrolases

**Figure 8:**
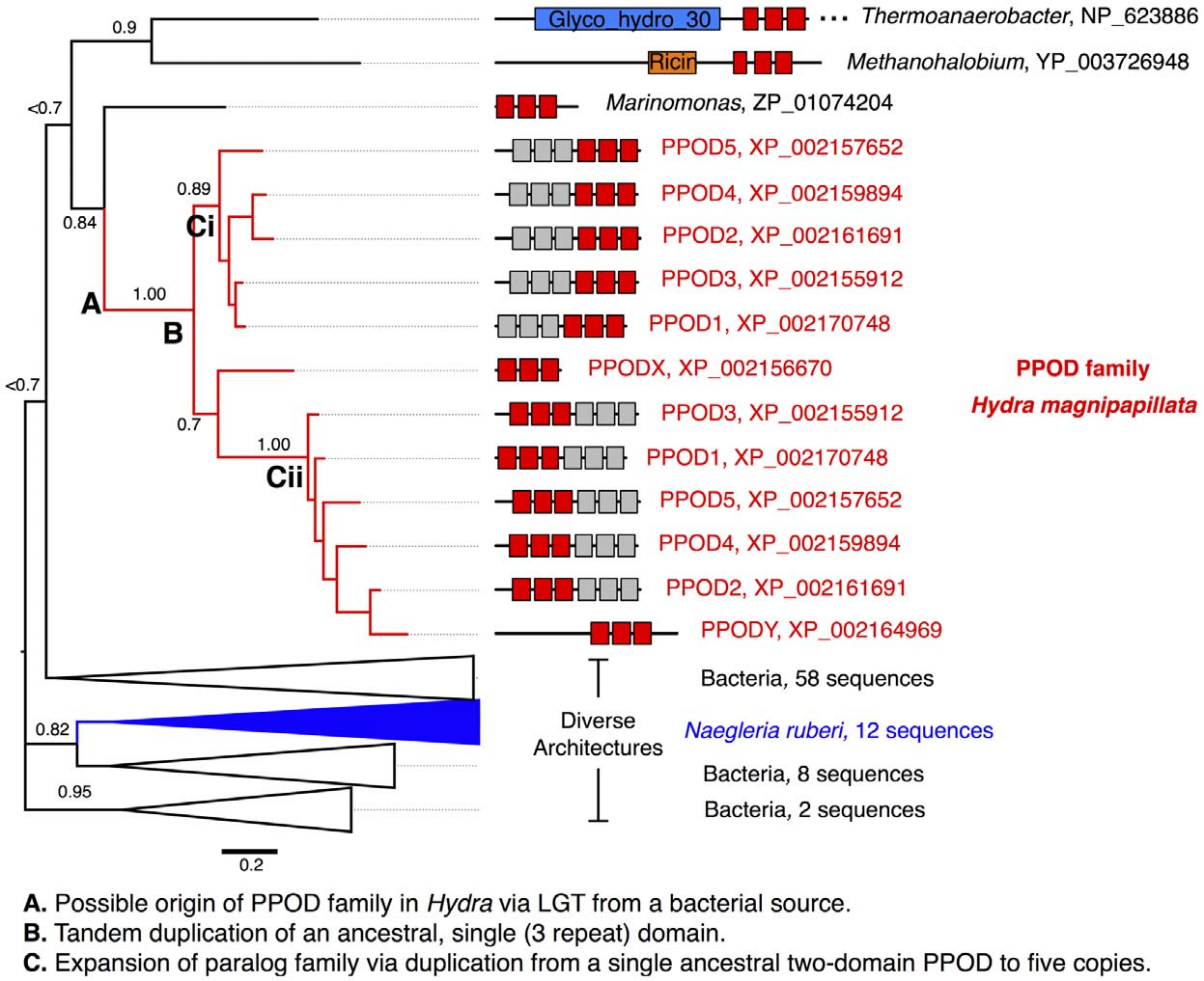
Evolutionary analysis of *Hydra* PPOD reveals a link to bacterial carbohydrate-binding proteins. A bayesian inference phylogenetic tree was generated for the *Hydra* PPOD family and the top-scoring related sequences retrieved by BLAST. The *Hydra* PPOD family forms a single clade restricted to *Hydra* and has expanded by tandem duplication of a single β-trefoil domain, followed by whole-gene duplications. The closest ancestral sequences are of bacterial origin. The most related sequences include additional domains such as glycosyl hydrolase domains and sugar-binding domains (e.g., ricin). Unlike most of the detected bacterial homologs, the closest outgroup of the *Hydra* PPOD clade (a sequence from *Marinomonas*) has a single β-trefoil domain, supporting its close relationship to the inferred ancstral PPOD domain. Posterior probabilities are indicated above the major clades. See Fig. S4 for an expanded version of this tree.

Proteins with similarity to PPODs were detected using BLAST (default parameters) with PPOD4 as a query. As expected, all known PPOD sequences were retrieved as well as several novel PPOD-related sequences from *Hydra magnipapillata,* whose genome has been sequenced [11]. Surprisingly, outside of the genus *Hydra*, most top-scoring proteins detected by BLAST were from bacterial species. For instance, a three-repeat protein (putative single domain β-trefoil) from *Marinomonas sp.* was found with an E-value of 2e-19 and 44% sequence identity to PPOD 4 (Fig. 8). The only set of eukaryotic sequences with scores comparable to those of the bacterial proteins were sequences from the amoeba, *Naegleria*. Eukaryotic fascin proteins were only detected in PSI-BLAST iteration two, and aligned with much lower sequence identity (21%). These results are consistent with a previous genome-wide analysis of *Hydra magnipapillata* which listed PPODs as candidate genes that may have entered the *Hydra* genome by horizontal gene transfer [11].

To characterize the relationship between PPODs and related proteins we carried out a phylogenetic analysis. A ‘domain-based’ alignment was generated by splitting PPODs into their first (repeats 1–3) and second (repeats 4–6) β-trefoil domains, and aligning the domains to the regions detected as most similar by BLAST. After removing redundant sequences and poorly aligned regions, the remaining regions of the alignment were selected to build a maximum-likelihood tree with PhyML (see Methods) [31]. In this tree (Fig. 8), the *Hydra* PPOD domain sequences form a single clade with perfect (1.00) bootstrap support. The topology of the tree suggests that the *Hydra* PPOD family arose from a single, ancestral, β-trefoil domain. The PPOD family is derived within *Hydra*, suggesting that the ancestral domain may have existed in or may have been transferred to an ancestral *Hydra* species. Interestingly, the most closely related non-*Hydra* sequence (*Marinomonas* PPOD-like domain) has a single-domain structure, which resembles the proposed ancestral PPOD prior to domain duplication. Based on the tree, the single-domain PPOD ancestor underwent an intra-gene, tandem duplication (labeled ‘B’), producing two sister clades Ci and Cii. Following this, successive whole-gene duplications including both domains produced PPODs 1 to 5 within clades Ci and Cii.

As illustrated in Fig. 8, the six-repeat architecture of PPODs is distinct from that of homologs found outside of *Hydra*. While a few bacterial PPOD-like proteins have single PPOD domains (Fig. S4), in most cases the PPOD domains are present in hydrolytic enzymes. Their association with cellulases, chitinases, numerous glycosyl hydrolases, and the lectin domain of the potent toxin ricin (Fig. S4) suggests that PPOD-like sequences may function as carbohydrate-binding modules. In fact, carbohydrate-binding is the most common function associated with β-trefoil domain families. PFAM’s “Trefoil” clan (http://pfam.sanger.ac.uk/clan/Trefoil), for instance, contains the domain families AbfB, Agglutinin, Ricin B_Lectin, and Toxin R_Bind_C, all of which are known to bind carbohydrates.

#### Comparative structural analysis reveals a 3-fold repeated lectin-like site in PPODs

**Figure 9:**
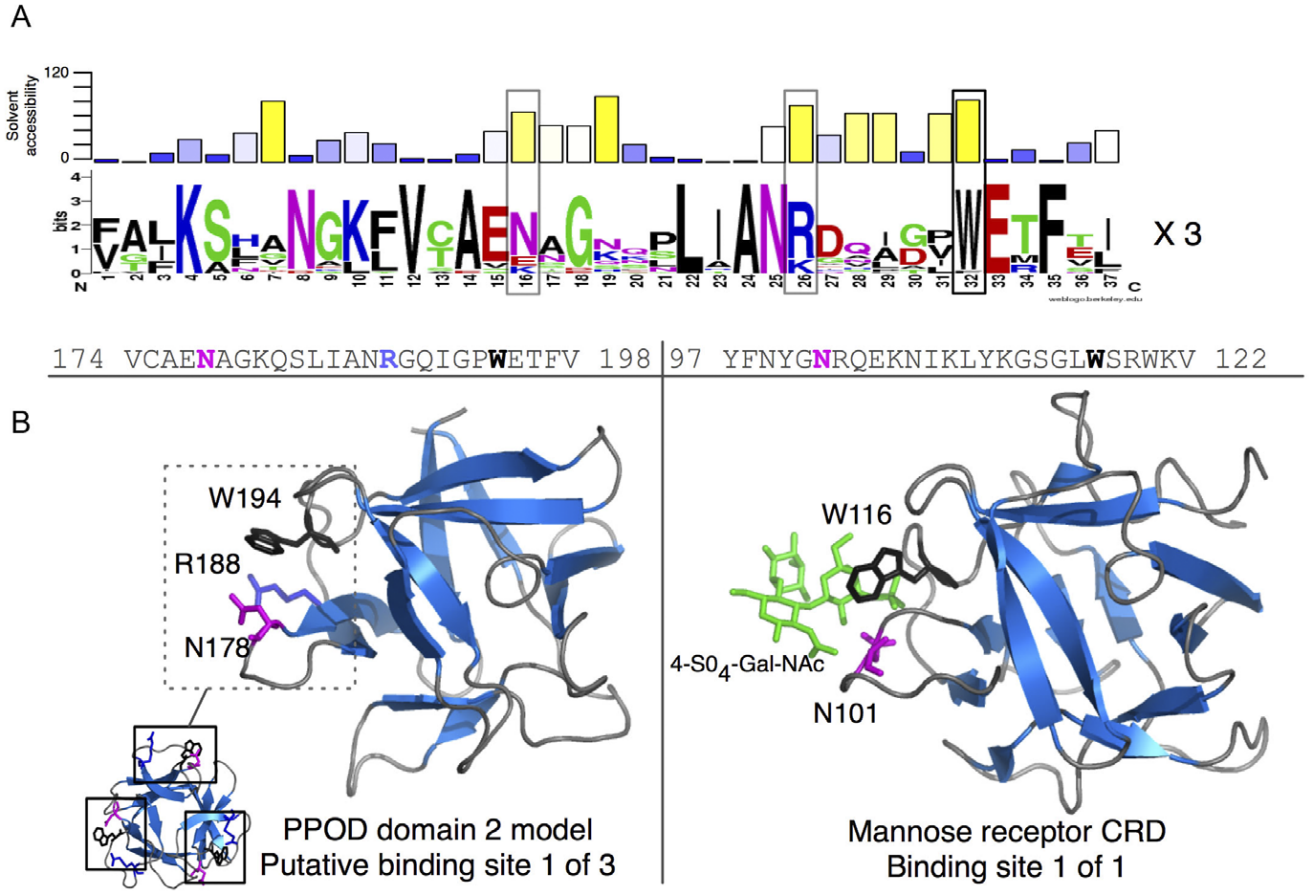
Structural modeling suggests a carbohydrate-binding function for PPOD. A) A sequence logo was generated by combining all repeats from the PPOD family into a single alignment. Above the sequence logo, the solvent-accessibility has been plotted as calculated from the binding-site associated repeat shown in (B). B) Left - a structural model of PPOD4 domain 2 with putative sugar-binding Trp residues highlighted in red. Right - Structure of the CRD from the mannose receptor bound 4-SO4-Gal-NAc (PDB ID 1FWV). The matching N and W residues are indicated in the alignment above, as well as an additional conserved residue (R188) that is also located close to the putative binding site.

The sequence conservation patterns of PPODs were examined within the context of the structural model to identify conserved surface residues that may be of functional importance. As the conservation profiles for each individual repeat were highly redundant, the repeats were combined into a single alignment and made into a sequence logo (Fig. 9A). Below the logo, the solvent accessibility has been plotted as calculated from the structural model in Fig. 9A. Residues that are both highly conserved and accessible represent likely functional sites. The top site that meets both of these criteria is the Trp residue found in the WEXF motif at the C-terminal end of each repeat (boxed in Fig. 9A). Trp is typically rare on the protein surface, with the exception of carbohydrate-binding sites where it is an overrepresented residue and can play a crucial role in C-H/π-stacking with the B-face of the pyranose ring [38]–[40].

Given a possible carbohydrate-binding function, we compared the PPOD structural model to other β-trefoil carbohydrate-binding structures. A striking similarity was observed when the PPOD model was aligned and compared to the β-trefoil domain of the mouse macrophage mannose receptor, a bifunctional lectin with C-type and β-trefoil domains [41]. The cysteine-rich β-trefoil domain is complexed in crystals with GalNAc-4-S0_4_. When the two structures are superimposed in the orientation shown (Fig. 9B), the Trp residue in PPODs (e.g., W281) matches the corresponding Trp residue (W116) in the carbohydrate recognition domain (CRD) of the mannose receptor that is in contact with the sulfated sugar. Another residue (N101), defined as an additional sugar-binding residue in the β-trefoil domain of the mannose receptor [41], is shared by PPODs (N265) in the same structural position (boxed in Fig. 9A). A third residue that is unique and highly conserved in the PPOD repeats but that is not found in the mannose receptor is R275. Given its conservation and proximity to W281 and N265, it is possible that R275 is an additional carbohydrate-binding residue, making an interaction with negatively charged sugars likely.

The β-trefoil domain of the mannose receptor CRD contains a single carbohydrate-binding site, while the PPOD model contains three such sites (Fig. 9B, bottom left). Interestingly, 3-fold multiplicity of carbohydrate-binding sites is a feature of numerous other β-trefoil lectins, and is thought to provide multivalent carbohydrate binding [42], [43]) and increase target affinity and/or selectivity [39]. Thus, we compared the PPOD model with the β-trefoil xylan-binding domain of CBM13 from *Streptomyces lividans,* which exhibits multivalent binding to three lactose molecules [44]. The three lactose-binding sites in CBM13 structurally align with the positions of the three Trp residues in the PPOD model (Fig. S5). The lactose moieties bound by CBM13 are in contact with a Trp and two Tyr residues (a conservative substitution for Trp in several sugar-binding sites) in equivalent positions to the three Trp residues in the PPOD model. Thus, structurally it appears that PPOD domains possess similar residues in similar positions when compared to sugar-binding residues of known β-trefoil lectins.

### PPOD has Lectin-like Haemagglutination Activity which is Inhibited by Chondroitin and Heparan Sulfates In Vitro

**Figure 10:**
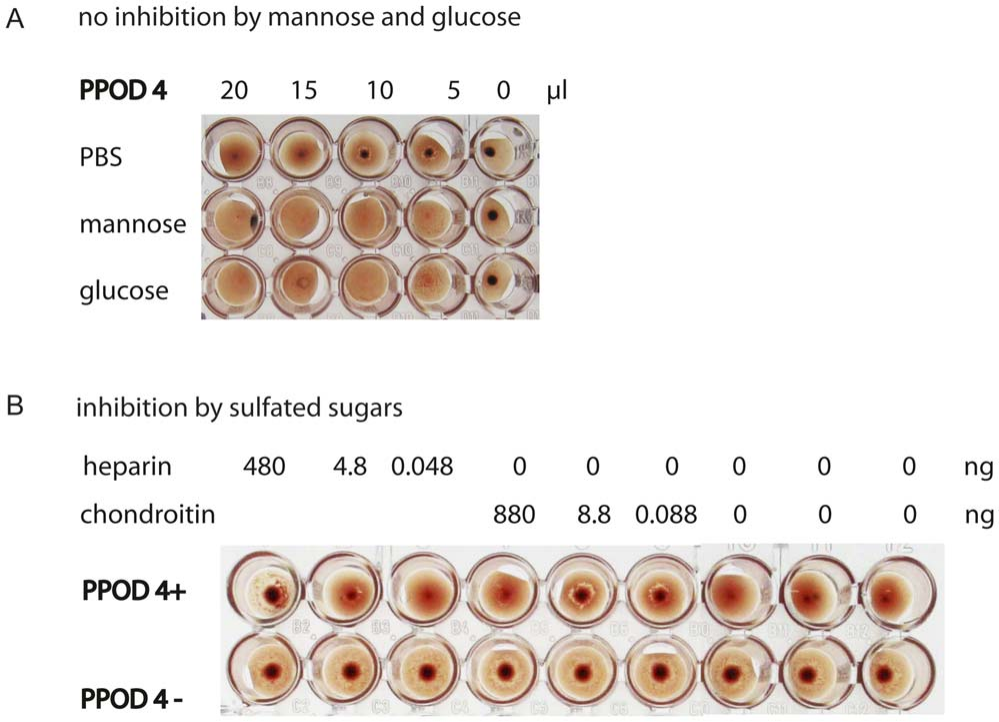
PPOD agglutinates erythrocytes. Haemagglutination assays with rabbit erythrocytes. A: Addition of increasing amounts PPOD4 protein agglutinates erythrocytes and prevents their sedimentation (dark dot at 0 µl PPOD4 indicates sedimentation of erythrocytes, which are not agglutinated). Addition of mannose or glucose does not prevent PPOD4 induced agglutination. B: Erythrocytes sediment in the absence of PPOD4 protein (lower row). Addition of PPOD4 prevents this due to agglutination. Addition of heparin or chondroitin (GalNAc-4-SO4) to PPOD prevents PPOD4 induced agglutination in a concentration dependent manner.

The structural similarity of PPOD family proteins to β-trefoil type lectins prompted us to test whether PPODs are agglutinins and can bind sugars. We performed haemagglutination assays using trypsin-treated, paraformaldehyde-fixed rabbit erythrocytes. Fig. 10A shows that agglutination of erythrocytes was achieved with 3 µg of His-tagged recombinant PPOD protein. To test whether sugars can inhibit PPOD-mediated agglutination we added different neutral sugars and derivatives to the agglutination reaction, namely D(+)mannose, D(+)galactose, α-methyl-D-mannopyranoside, n-dodecyl-β-D-maltoside, D(-)mannit, D(-)fructose, N-acetyl-D-glucosamine, N-acetyl-D-galactosamine, sucrose and lactose. None of these was able to inhibit agglutination. We then attempted to inhibit agglutination with heparin (a polyanion containing sulfated GlcNAc and (sulfated) glucuronic and iduronic acids from bovine lung) and chondroitin (a polyanion of glucuronic acid and sulfated GalNAc from whale cartilage). The results in Fig. 10B show that both compounds inhibited PPOD-mediated agglutination in a concentration dependent manner. Thus PPODs appear to be soluble agglutinins capable of binding sulfated glycosaminoglycans.

## Discussion and Conclusions

### The Freshwater Polyp *Hydra* is Covered by a Cuticle

The present study shows that *Hydra* polyps are covered by an extracellular cuticle with a complex layered structure consisting of several proteins and glycosaminoglycans. Although earlier EM studies on chemically fixed tissue have generally shown a thin 100–200 nm sheet covering the ectodermal surface of *Hydra,* cryo-based EM indicates that this extracellular structure is larger and considerably more complex than previously known, being 1.5 µm thick and having a clear substructure consisting of five distinct layers of fibrous material all of which are PAS positive (Figs. 1, 2). This finding suggested that these fibrous structures may consist of complex carbohydrates and biochemical analysis of the isolated cuticle layer showed the presence of chondroitin and chondroitin sulfate chains (Fig. 3).

The cuticle appears to be an extracellular sheet, very closely opposed to the ectodermal surface as seen in cryo-fixed samples. However, it is not well preserved by conventional chemical fixation procedures. For example, in PFA- or Lawdowsky-fixed preparations, and even after fixation with glutaraldehyde and osmium tetroxide, the cuticle is usually collapsed, fragmented or detached from the ectodermal surface (note that addition of ruthenium red during fixation improves the preservation of the cuticle: see Fig. S1).

The cuticle is synthesized by ectodermal epithelial cells. These epithelial cells have numerous secretory vesicles under the apical surface and they stain strongly with the PAS reagent suggesting that they are the source of the PAS reactive material in the cuticle (Figs. 1, 2). These secretory vesicles also contain PPOD, one of the prominent protein components of the cuticle (Figs. 5, 6). Nevertheless, it is unclear how the complex layered structure of the cuticle is produced, since secretion of vesicles containing cuticle components occurs at the base of the cuticle (Fig. 6B, C). Do these components self-organize following secretion? Given that PPOD has haemagglutination activity which is inhibited by glycosaminoglycans, it could play a significant role in organizing glycosaminoglycan chains in the cuticle.

### Possible Functions of the Cuticle

The complex cuticle described here for *Hydra* is secreted directly by the ectodermal epithelial cells. It has ultrastructural similarity with the glycocalyx of some amoebae and also with that of mammalian epithelia lining the lumen of vessels, intestine and organs. In other cnidarians similar structures have been described by conventional EM, e.g. in *Cryptohydra thieli* and in *Coryne tubulosa* and *Syncoryne tenella* [45], [46] However, since the cryo-fixation method used here preserves the ultrastructure much better, it is difficult to compare our description of the *Hydra* cuticle with these earlier descriptions of the extracellular surface of other cnidarians.

The *Hydra* cuticle covers the entire surface of the polyp with the exception of the sensory cilia (cnidocil) of nematocytes mounted in tentacles and along the body column. The cuticle almost certainly provides physical protection to the cell surface, since it is the only structure separating the cell membrane from the environment. In particular, the cuticle may protect the cell surface by forming an acellular layer which can be sloughed off on demand. It could also be involved in protecting the cell membrane from osmotic lysis in the freshwater environment surrounding *Hydra* polyps. It is negatively charged, due to the high concentration of sulfated glycosaminoglycans and presumably binds cations thus increasing the ion concentration locally at the surface of the epithelial cells. Finally, the cuticle could regulate the composition of the surface microbiome, e.g. by forming a physical barrier to bacteria or by binding anti-bacterial peptides and regulating the populations of bacterial symbionts co-existing with *Hydra* polyps [47]. One such symbiont, *Curvibacter sp*, was identified and its genome sequenced as part of the *Hydra* genome project [11]. In addition to influencing the bacterial growth on its surface, *Hydra* also has the ability to slough off the fragile cuticle layer c5 when massive bacterial overgrowth occurs (data not shown).

### PPOD has Two β-trefoil-domains with Similarity to Known Lectins

The experiments reported here demonstrate that PPOD, a major component of the cuticle, has structural similarity to lectins. The four PPOD proteins in *Hydra* have highly similar sequences of roughly 270 aa with two β-trefoil domains. Each domain consists of three highly similar repeats of about 40 amino acids (Fig. 7A). In particular, Trp residues occur at identical positions in each repeat. Bioinformatic analysis indicates that these conserved Trp residues lie within a pocket on the surface of the β-trefoil domains (Fig. 9). Such solvent-exposed Trp residues are uncommon in proteins but are found in sugar-binding motifs of several lectins and CBMs. The sequence within the putative sugar-binding motif of PPODs is highly similar to the sequence in the cysteine-rich domain of the mammalian mannose receptor (40% sequence identity). This β-trefoil domain of a mammalian lectin also contains a solvent-exposed Trp residue at the same position as the Trp residues in PPOD and this residue has been shown to be involved in binding sulfated sugars [41], [48]. Further signature residues are also conserved. While the mannose receptor contains only one β-trefoil sugar-binding site, the PPOD proteins are predicted to have three sugar-binding sites within each of the two β-trefoil domains. Thus PPOD proteins are predicted to be multivalent and hence could crosslink different glycosaminoglycan chains together.

The predicted sugar-binding function of PPOD proteins was investigated with haemagglutination experiments. PPOD proteins clearly induced agglutination of red blood cells at low protein concentrations (Fig. 10). This result indicates multivalency. While numerous neutral monosaccharides including GlcNAc and GalNAc did not affect this agglutination activity, the sulfated polysaccharide chains chondroitin sulfate and heparin/heparan sulfate both inhibited agglutination making a specificity for charged sugars likely. However, a primarily ionic interaction with suitably positioned sulfate groups cannot be completely excluded, as for example discussed for bindin, a receptor for sulfate esters present on sea urchin sperm and acting as adhesion molecules [49], [50].

Interestingly, SWT proteins present in the cuticle layer (Fig. 4A) may also have sugar binding activity. Structural predictions indicate similarity of the SWT proteins to a β-sandwich structural domain found in lectins of leguminous plants and in several extracellular proteins, including the pentraxin C-reactive protein (unpublished observations). It will be very interesting to test these predictions in the future and to investigate the localisation of SWT-proteins within the cuticle in comparison with PPOD proteins.

### Evolutionary Origin of PPOD Proteins and the *Hydra* Cuticle

PPOD proteins are represented in genomic and EST sequence databases of all *Hydra* species examined [12], but are not found in sequence databases for any other cnidarians [11], nor in any metazoan sequence databases (Fig. 8). In particular, the EST databases for the closely related marine hydrozoans *Clytia*, *Cladonema* and *Hydractinia* lack PPOD sequences. Similarly, the *Hydra* cuticle appears to be a unique structure although there is less extensive comparative data for other cnidarians than is the case for PPOD sequences. Nevertheless, one can speculate that the *Hydra* cuticle evolved modifications during the transition from the marine to the freshwater environment. For example, the lectin-like activity of PPOD proteins could mediate binding to and structural re-organization of glycosaminoglycans already present on the surface of an ancestral *Hydra*. In this regard it would be interesting to investigate the cuticle structure of the freshwater hydrozoan medusa *Craspedacusta*, which is phylogenetically distant from *Hydra* and entered the freshwater environment independently of *Hydra* [51]. The absence of PPOD sequences in the recently published EST database for *Craspedacusta* [52] suggests that PPOD is not essential for the marine to freshwater transition and that the appearance of PPOD sequences in *Hydra* is a taxon-specific event.

The apparent presence of PPOD genes only in *Hydra* and in several bacterial genomes has led to the suggestion that they entered the *Hydra* genome by horizontal gene transfer [11]. Although horizontal gene transfer is expected to be a rare event, Moran et al. have recently described multiple examples of horizontal gene transfer of a pore-forming toxin gene between metazoan species [53]. Based on these results, they have argued that genes which can (a) directly carry out a biochemical function and (b) be immediately beneficial to the host may be favored for horizontal gene transfer. PPODs appear to fulfill these two critieria. PPODs are agglutinins and could bind to and presumably organize glycosaminoglycans already present on the surface of an ancestral *Hydra*. This PPOD ancestor could then have been amplified or diversified through successive rounds of gene duplication, as illustrated in Fig. 8, to generate a *Hydra*-specific gene family.

## Supporting Information

Figure S1 **Hypertonic salt wash removes cuticle layer c5.** Cuticle of (A) control *Hydra* and (B) *Hydra* treated for 5 minutes with 200 mM NaCl. Polyps were chemically fixed with glutaraldehyde and osmium tetroxide in the presence of ruthenium red to stabilize cuticle structures. Control animal shows intact cuticle layers c1 to c5. NaCl treated *Hydra* shows almost complete loss of cuticle layer c5 and partial disruption of cuticle layer c4; layers c1–c3 are less affected. Scale bar 500 nm.
(JPG)

Figure S2 **PPOD antibody on Western blot.** 3 ng or 6 ng of recombinant His-tagged PPOD protein after purification from E.coli lysates and a lysate from *Hydra* cells were separated in SDS-PAGE, immunoblotted and probed with anti-PPOD4 antibody. Both, recombinant and endogenous PPOD4 are recognised by the antibody at the predicted molecular mass of 27 kDa.
(PDF)

Figure S3 **PPOD does not have peroxidase activity.** Recombinant PPOD4 produced in *E. coli* and *Hydra* lysate were separated on semi-native LDS-PAGE gels and probed with anti-PPOD4 antibody (left hand side) or subjected to in gel peroxidase assay. PPOD4 does not have peroxidase activity. A peroxidase activity is present in the *Hydra* lysate, but migrates at a different position from PPOD4.
(PDF)

Figure S4 **Expanded phylogeny and domain architectures of PPOD-related proteins.** A bayesian inference phylogenetic tree was generated of the *Hydra* PPOD family and the top-scoring related sequences retrieved by BLAST, encompassing a total set of 95 beta-trefoil domain sequences. The *Hydra* PPOD family is indicated in red, bacterial sequences in black, and *Naegleria* sequences in blue. The domain architectures of the full-length proteins are shown on the right of the tree, and predominantly include carbohydrate processing enzymes and binding domains. A condensed version of this tree is shown in Fig. 8.
(PDF)

Figure S5 **Comparison of PPOD structural model (left) with the xylan binding domain from** ***Streptomyces lividans*** **xylanase (right).** The PPOD structural model contains three putative binding sites in similar positions to the *S. lividans* xylan binding domain, which exhibits multivalent carbohydrate-binding activity.
(PDF)
