# Supplementary figures and images for: Horizontal Gene Transfer Contributed to the Evolution of Extracellular Surface Structures: The Freshwater Polyp Hydra Is Covered by a Complex Fibrous Cuticle Containing Glycosaminoglycans and Proteins of the PPOD and SWT (Sweet Tooth) Families
Source: PLoS One. 2012 Dec 27;7(12):e52278. doi: 10.1371/journal.pone.0052278 (PMC3531485; doi:10.1371/journal.pone.0052278)

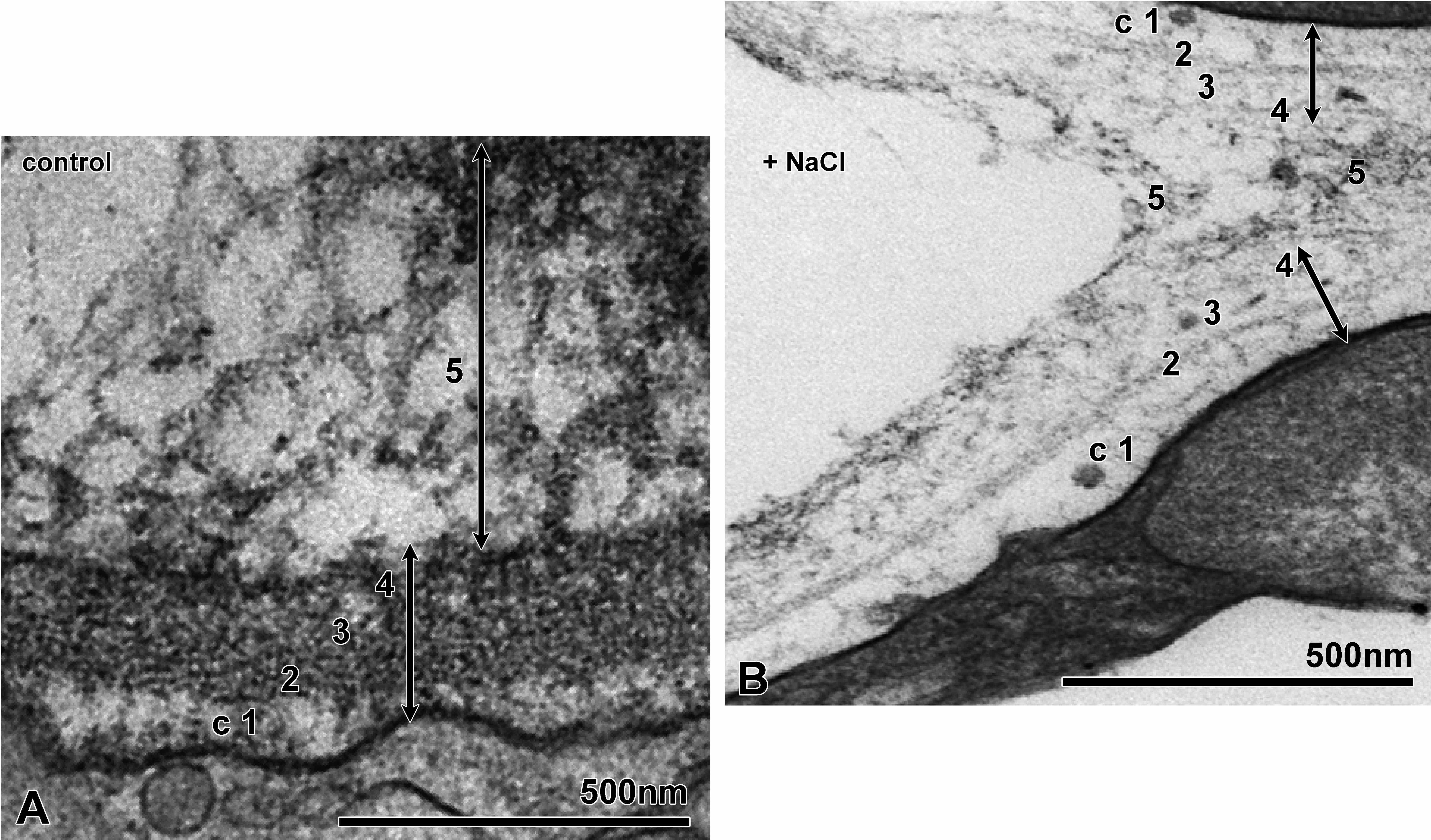

Supplement: Figure S1 — Hypertonic salt wash removes cuticle layer c5. Cuticle of (A) control Hydra and (B) Hydra treated for 5 minutes with 200 mM NaCl. Polyps were chemically fixed with glutaraldehyde and osmium tetroxide in the presence of ruthenium red to stabilize cuticle structures. Control animal shows intact cuticle layers c1 to c5. NaCl treated Hydra shows almost complete loss of cuticle layer c5 and partial disruption of cuticle layer c4; layers c1–c3 are less affected. Scale bar 500 nm. (JPG) [file pone.0052278.s001.jpg]

Fig. S2: Anti-PPOD Antibody Test

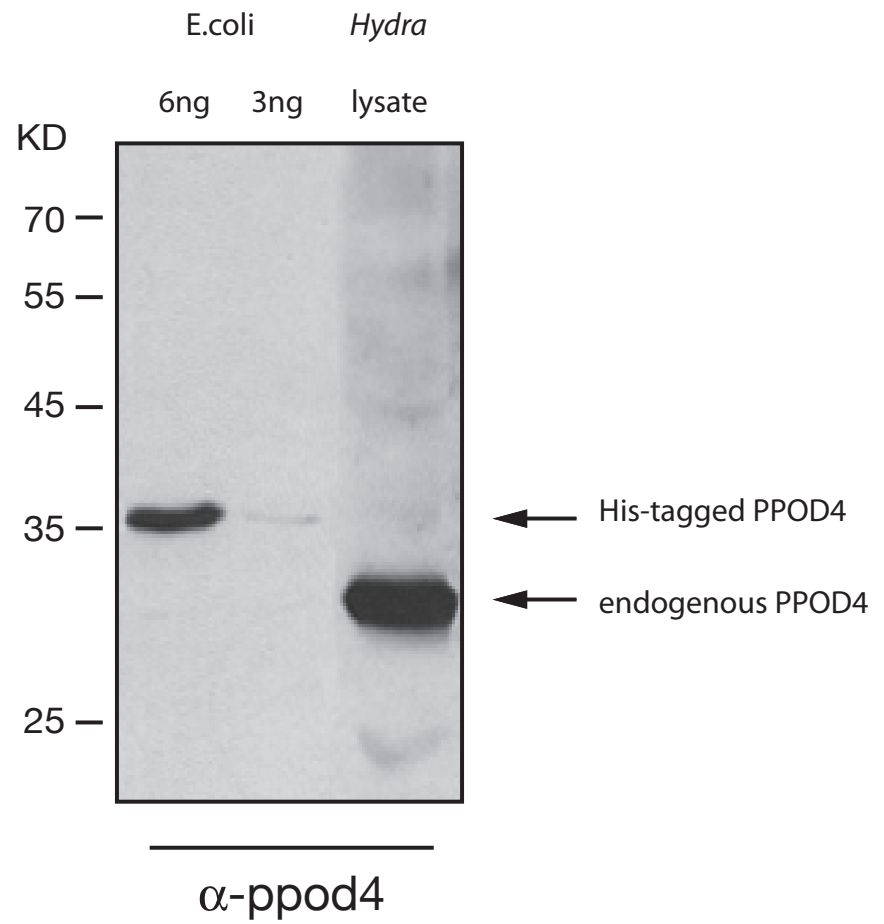

Supplement: Figure S2 — PPOD antibody on Western blot. 3 ng or 6 ng of recombinant His-tagged PPOD protein after purification from E.coli lysates and a lysate from Hydra cells were separated in SDS-PAGE, immunoblotted and probed with anti-PPOD4 antibody. Both, recombinant and endogenous PPOD4 are recognised by the antibody at the predicted molecular mass of 27 kDa. (PDF) [file pone.0052278.s002.pdf]

Fig. S3: Peroxidase Assay

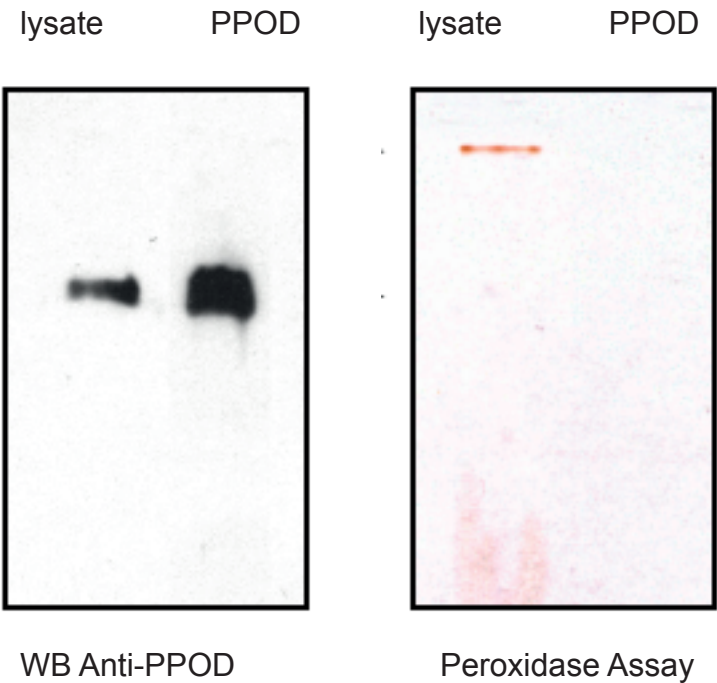

Supplement: Figure S3 — PPOD does not have peroxidase activity. Recombinant PPOD4 produced in E. coli and Hydra lysate were separated on semi-native LDS-PAGE gels and probed with anti-PPOD4 antibody (left hand side) or subjected to in gel peroxidase assay. PPOD4 does not have peroxidase activity. A peroxidase activity is present in the Hydra lysate, but migrates at a different position from PPOD4. (PDF) [file pone.0052278.s003.pdf]

Fig. S5: PPOD Comparative Structural Model

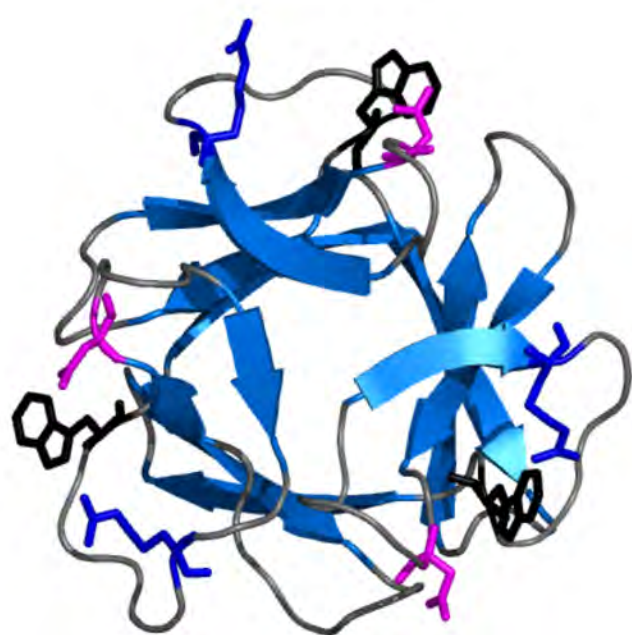

PPOD-model

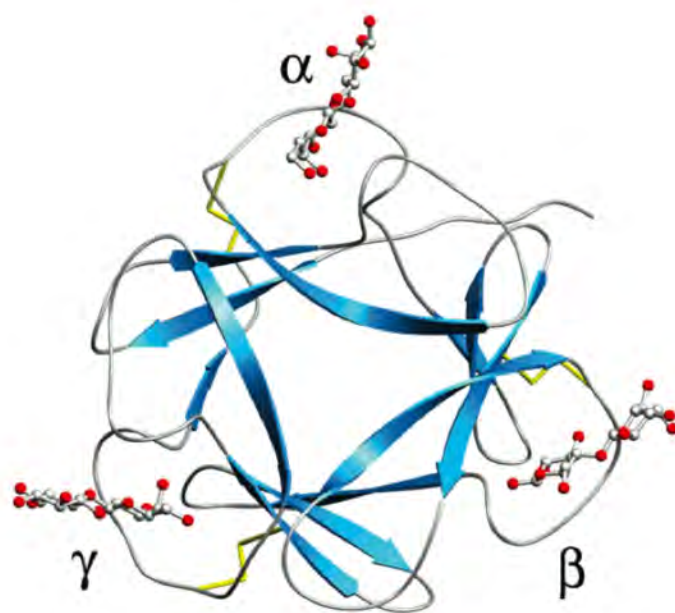

Xylanase 10A *Streptomyces lividans*

Supplement: Figure S5 — Comparison of PPOD structural model (left) with the xylan binding domain from Streptomyces lividans xylanase (right). The PPOD structural model contains three putative binding sites in similar positions to the S. lividans xylan binding domain, which exhibits multivalent carbohydrate-binding activity. (PDF) [file pone.0052278.s005.pdf]
