# Supplementary material for: Horizontal Gene Transfer Contributed to the Evolution of Extracellular Surface Structures: The Freshwater Polyp Hydra Is Covered by a Complex Fibrous Cuticle Containing Glycosaminoglycans and Proteins of the PPOD and SWT (Sweet Tooth) Families
Source: PLoS One. 2012 Dec 27;7(12):e52278. doi: 10.1371/journal.pone.0052278 (PMC3531485; doi:10.1371/journal.pone.0052278)

Fig. S4: Phylogeny of PPOD-related Domains

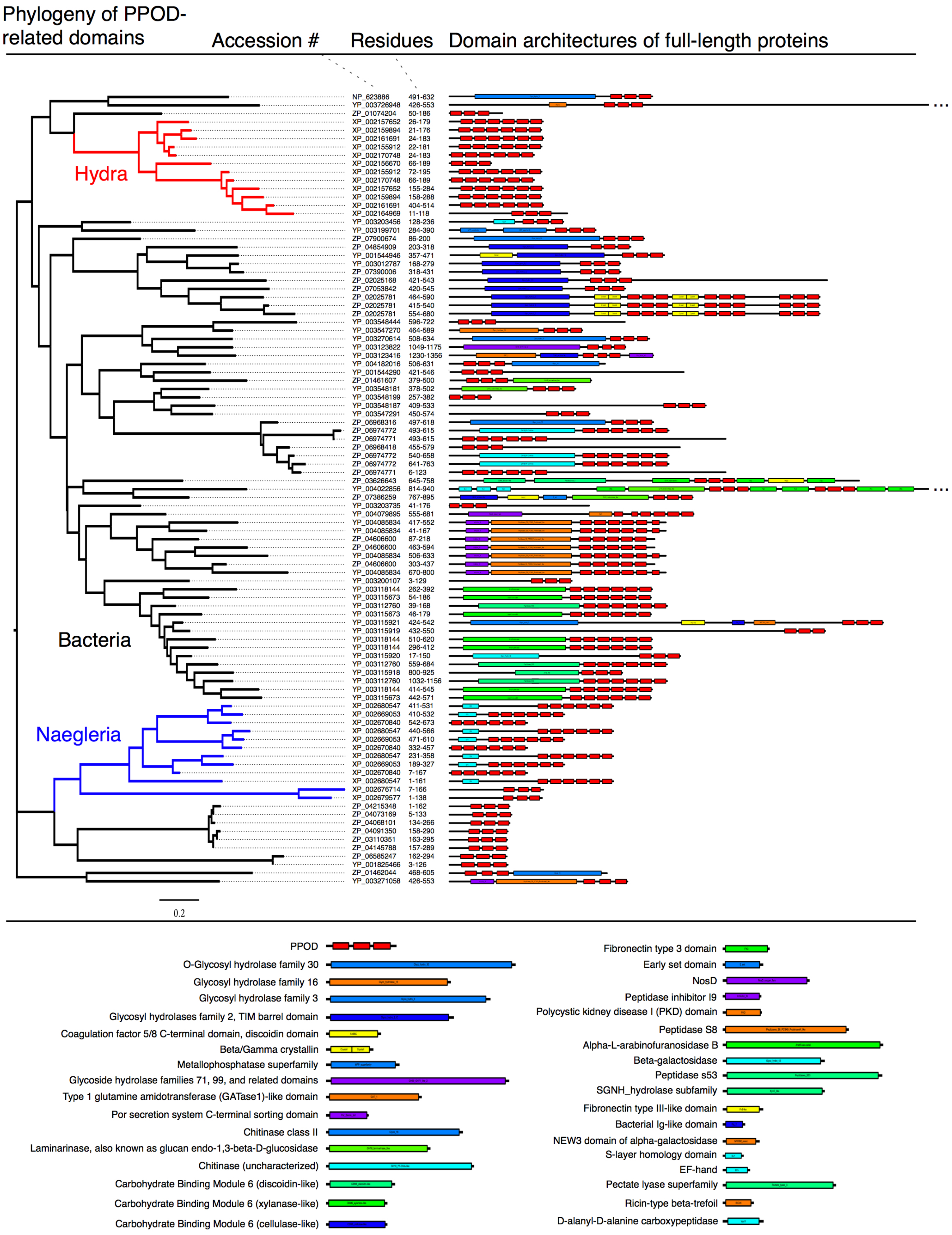

Supplement: Figure S4 — Expanded phylogeny and domain architectures of PPOD-related proteins. A bayesian inference phylogenetic tree was generated of the Hydra PPOD family and the top-scoring related sequences retrieved by BLAST, encompassing a total set of 95 beta-trefoil domain sequences. The Hydra PPOD family is indicated in red, bacterial sequences in black, and Naegleria sequences in blue. The domain architectures of the full-length proteins are shown on the right of the tree, and predominantly include carbohydrate processing enzymes and binding domains. A condensed version of this tree is shown in Fig. 8. (PDF) [file pone.0052278.s004.pdf]
